# Vertical Variability in morphology, chemistry and optical properties of the transported Saharan air layer measured from Cape Verde and the Caribbean

**DOI:** 10.1098/rsos.231433

**Published:** 2024-11-06

**Authors:** Sudharaj Aryasree, Konrad Kandler, Natalie Benker, Adrian Walser, Anne Tipka, Maximillian Dollner, Petra Seibert, Bernadett Weinzierl

**Affiliations:** ^1^ Institute of Applied Geosciences, Technical University of Darmstadt, Darmstadt 64287, Germany; ^2^ University of Vienna, Faculty of Physics, Aerosol Physics and Environmental Physics, Vienna 1090, Austria; ^3^ Dynamic Biosensors, Munich, Germany; ^4^ International Data Centre, Comprehensive Nuclear-Test-Ban Treaty Organization (CTBTO), PO Box 1200, Vienna 1400, Austria; ^5^ University of Natural Resources and Life Sciences (BOKU), Institute of Meteorology and Climatology, Vienna, Austria

**Keywords:** Saharan dust transport, dust mineralogy, silicate/sulphate mixing, particle settling velocity, single scattering albedo, dust imaginary refractive index

## Abstract

The structural properties of the Saharan air layer (SAL) including chemical, morphological and optical properties were measured during the Saharan Aerosol Longrange TRansport and Aerosol Cloud interaction Experiment (SALTRACE- June/July 2013). Flight measurements were done from Cape Verde and the Caribbean. Changes happening with the chemical composition, mixing, shape and absorption of aerosol single particles (particle diameter range 0.5–3.0 µm) inside SAL during its transport are detailed. Dust-dominated SAL (relative number abundance >90%) and generally low mixing (<1% with sea-salt and sulphates) are observed at both locations. The change in shape (determined as aspect ratio (AR)) after transatlantic transport was statistically not significant. The iron oxide fraction, important for light absorption, contributed 6.0–6.8% to SAL dust. A lower amount of Fe oxides was observed in transported SAL, especially for the size range 0.5–1.5 µm. This reduction in Fe oxide content resulted in a 4% decrease (0.0046–0.0044) in dust imaginary refractive index and a 1% decrease in single scattering albedo (0.802–0.809) at 520 nm. Our work suggests including the size distribution of iron oxides and their particular behaviour in future experiment/model studies.

## 1. Introduction

Mineral dust particles are transported from various arid/semi-arid regions to different geographical locations. The seasonal transport of Saharan dust to the Caribbean is highly important because of the direct and indirect climate effects of mineral dust particles, modification of atmospheric temperature profiles thereby altering the atmospheric circulations, and impact on precipitation patterns and cloud distributions [1–3]. Long-term measurements conducted over the Caribbean Islands [1] since the 1960s have provided a valuable dataset indicating the seasonal variations of transported dust with a summer maximum and winter minimum. Remote-sensing retrievals, as well as modelling studies, have corroborated well with *in situ* measurements [4–6] explaining the process of dust transport from active sources over the Sahara above the trade wind inversion in an elevated layer named the Saharan air layer (SAL) [7,8]. Over the Caribbean, this dust is deposited by dry and wet deposition, the rates of which strongly depend on the vertical dust concentration.

Various field campaigns conducted in North Africa have led to an improvement in the understanding of dust mobilization processes and desert dust characteristics, including size, shape, mineralogy, emission and transport, redistribution and deposition [9–12]. The most notable among them was conducted during the summer months (May–July) as part of the African Monsoon Multidisciplinary Analyses Program (AMMA [13]) and Saharan Mineral Dust Experiment (SAMUM-1, SAMUM-2 [14,15]). While AMMA measurements are devoted to an interdisciplinary study of the West African dust emission processes, atmospheric composition variability, and thereby effects, SAMUM-1 and SAMUM-2 are dedicated to an extensive characterization of mineral dust in the vicinity of the Sahara and in the outflow region of African dust in the Cape Verde (CV) region. Individual measurements conducted over the Caribbean during the Puerto Rico Dust Experiment (PRIDE [16]) characterized the properties of airborne mineral dust transported over the tropical North Atlantic and the Caribbean. Recent measurements from the Eastern Atlantic have confirmed the presence of coarse/giant particles (>20 µm) inside SAL. These particles were eventually transported and deposited across the Atlantic Ocean [17,18].

All these measurements provide valuable data in the field of dust transport and its associated processes. However, uncertainties remain regarding the changes in the physical, chemical and optical properties of dust after transatlantic transport. A recent study [19] has shown that the uncertainties in the values of the North African dust imaginary refractive index account for an overestimation of their dust absorption aerosol optical depth by a factor of 2. The imaginary refractive index of dust is mainly determined by the particle mineralogy, or more precisely, the iron content present in the total dust that exists as structural iron, free iron or oxyhydroxides (hematite/goethite). The latter is the main component which absorbs solar radiation [20]. Soils from various arid locations in Asia, North Africa and other dust source regions in the world have different total iron contents. The content varies from 3.6% to 6.6% with the highest values found in Sahel [20]. A significant fraction of this iron content is light absorbing. These regional differences in iron contents have a major impact on the dust-single scattering albedo (SSA) values. Experimental studies have observed a linear decrease in SSA when the iron oxide content exceeds 3% [20]. A recent study by Di Biagio *et al*. [20] compiled a dataset for the total iron content and its oxide percentage over 11 source regions of the Sahara, three in the Middle East, two major dust sources in Asia and three regions in America. They found that the iron oxides over the Saharan region accounted for 35–50% of the elemental iron with haematite being the major iron oxide in Southern Africa while goethite dominated in the major Northern source regions. These variations in the iron content across different regions influence dust absorption, which is reflected in the values of the complex refractive index (*k*) and SSA. These values, in turn, affect the sign of the dust radiative effect. Hence, a better constraint of these regional differences in the optical properties of dust is necessary to reduce the uncertainty in the estimation of dust direct radiative effects (DREs) by modelling or remote-sensing observations [21].

Other critical parameters that affect the estimates of dust radiative forcing are the particle size and morphology (shape) distribution during the emission and how they change during short-long-range transport. In this study, we focus on changes in morphology, while size distribution changes are discussed elsewhere [22]. The particle morphology changes with transport have been studied previously and explained by gravitational settling, turbulence and ageing when particles act as cloud-condensing nuclei or ice nuclei (CCN and INP) [23,24]. Dust asphericity affects the drag force of a particle, thereby decreasing the settling velocity of the particle compared with its spherical counterpart [25,26]. Recent studies in dust modelling have shown that after accounting for the asphericity in the calculation of gravitational settling, dust lifetime was increased by 20%. Not accounting for this increase in dust lifetime could create biases in models, especially in the representation of coarse-mode dust. Understanding this complex interaction of dust is challenging and often requires comprehensive, coordinated measurements and state-of-the-art modelling to obtain a clear or concise picture of dust transport and its effects on the Earth’s system [22].

The SALTRACE campaign was conceived to understand the physical, chemical and radiative characteristics and modification of atmospheric dust during its transatlantic transport. The measurement campaign was conducted from spring 2013 through summer 2014 to characterize the dust flow into the Caribbean using ground-based, airborne and remote-sensing observations before and after the SAL transport. The present work was carried out in the context of SALTRACE and focused on airborne *in situ* data from the June to July 2013 period. SALTRACE measurements provided a unique dataset of the microphysical, chemical and optical properties of the SAL with distinct dust events, a low dust loading period and a high dust loading period after a tropical storm. During the first dust event on 11–13 June 2013, measurements were conducted over 15–23°W, 14–17°N concentrating on the airmass advecting towards the coastal regions of West Africa and Eastern Atlantic. On the following days (20–22 June), intensive experiments were done to study the characteristics of this transported dust above the marine boundary layer (BL) in the Caribbean. The Lagrangian experiment conducted [22] during this period reported a decrease of AOD (500 nm) by a factor of 2 and coarse-mode number concentration decreased by 10% inside the SAL after its transport. The results of the Lagrangian experiment have been published in the SALTRACE overview paper [22]. The last dust event studied here was observed by the end of the campaign from 10 to 11 July 2013 after the passage of tropical storm Chantal. The objective of our study was to provide a comprehensive measurement of dust mineralogy, their morphology and dust optical properties from two locations, first near the source (CV, Eastern Atlantic) and second after the transatlantic transport (Caribbean, North Atlantic). The campaign period also provided an opportunity to study the SAL characteristics before and after the passage of tropical storm Chantal [22] (8–9 July 2013, https://www.nhc.noaa.gov/data/tcr/AL032013_Chantal.pdf).

## 2. Methods

### 2.1. Field campaign

**Figure 1.**
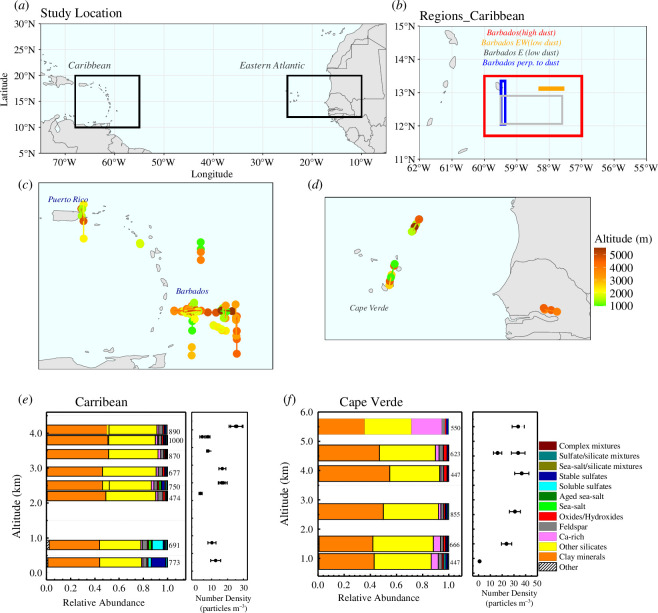
Flight details and the single-particle composition at various altitudes over CV and Caribbean.(* a*) Map with boxed locations where the flight measurements were done; (*b*) locations studying dust variability during high dust and low dust loading conditions near Barbados. (*c,d*) The mean position of sampling in the Caribbean and the Eastern Atlantic with their altitude given in colour code; (***e***) left: Relative abundance of major aerosol classes identified (in the Caribbean) at different altitudes extending from 300 m to 4.5 km each bar represents one sample/flight section, the number of particles analysed is given beside the bars; *right:* The median particle number density for the respective altitudes averaged over the sampling time with standard deviation as error bar**;** (*f*) left and right: Same as (*e)* but over CV at different altitudes.

Aerosol particle sampling was performed onboard the Falcon aircraft platform [22] using a mini impactor sampler [27], during the SALTRACE campaign. The focus of this campaign was to achieve an atmospheric column closure experiment, combining ground-based, airborne *in situ* and remote-sensing observations. Measurements were carried out at Barbados (the main supersite), Puerto Rico and Cabo Verde during June and July 2013 (figure 1*a*). Here, we focus mainly on the airborne *in situ* measurement of particles collected from the main sites of CV and the Caribbean. The flight measurements near the Eastern Atlantic were done on 11, 12 and 17 June 2013, while the 20–22 June sampling studied the dust variability during ‘high dust loading’ conditions [22] with extended east–west and north–south sampling flights near Barbados (figure 1*b*, table 1). The East of Antigua and Puerto Rico measurements were covered in the 30 June and 1 July measurements. The passage of tropical storm Chantal (8–10 July) was accompanied by ‘low dust loading’ in the Caribbean and the SALTRACE campaign ended on 11 July 2013.

**Table 1.** Acronyms of study regions used in the result section. The regions near Barbados are also marked in figure 1*b*.

acronyms	region defined
CV SAL	SAL in the Eastern Atlantic (high dust conditions) (figure 1*d*)
CV BL	BL in the Eastern Atlantic (high dust conditions) (figure 1*d*)
Bar SAL	SAL in the vicinity of Barbados high dust conditions) (figure 1*b*)
BarBL	BL in the vicinity of Barbados (high dust conditions) (figure 1*b*)
BaPSAL	East of Barbados perpendicular to dust flow (low dust) (figure 1*b*)
BaESAL	East of Barbados, SAL (low dust) (figure 1*b*)
BaEWSAL	East–West of Barbados, SAL (low dust condition) (figure 1 b)
PRBL	Puerto Rico and East of Antigua BL (figure 1*c*)
PRSAL	Puerto Rico and East of Antigua SAL (figure 1*c*)

The ‘multi-mini’ impactor [27,28] collected particles in two stages with diameters of approx. 0.1–1.0 and 1–3 µm. Owing to the efficiency of the Falcon aircraft common inlet [29] through which the air is sampled inside the measurement cabin, the upper cut-off size at a collection efficiency of 50% is defined as roughly 3 µm. The sampling time was approx. 5–10 min (the start and end of each sampling session are represented by the respective line segments with solid circles, in figure 1*c,d* at various altitudes). Particles were collected on a black carbon substrate glued to a metal stub. After collection, the samples were stored in airtight boxes under low humidity conditions until further analysis in the lab. Blank substrates (samples) were collected and stored along with the samples. After inspection, it was found that the blank substrates were devoid of particles which is interesting for this study. And the particles that were present were classified into class ‘artefacts’ and removed from further analysis. Electronic supplementary material, table 1a with sampling details is provided in the electronic supplementary material §1. Electronic supplementary material, table S1b provides the sample numbers used to study the variability of dust from various locations as denoted in figure 1*a–c*.

### 2.2. Electron microscopy analysis

The chemical composition and single-particle characteristics of aerosols were studied using a scanning electron microscope coupled with energy-dispersive X-ray spectroscopy (SEM-EDX) to obtain information on their size, shape and chemical composition. Sample analysis was performed automatically by the software-controlled (EDAX/AMETEK GENESIS 5.231) electron microscope [30]. Automated SEM analysis was performed under high vacuum conditions, with an accelerating voltage of 12.5 kV. Therefore, the particles were considered as dry during the time of analysis. Back-scattered images with a resolution of 1024 × 800 and a pixel size of 36 nm/pixel were used to get information on particles >0.5 µm projected area diameter (PAD). Smaller particles (<0.5 µm) were not considered in this study due to their low *S*/*N* ratio for this particle substrate combination. This method is efficient when the concentration of particles collected is less than a few nanograms when methods like X-Ray Diffraction (XRD) or X-Ray Fluorescence (XRF) are inefficient, where the latter needs at least the total mass in milligrams. During our study, the total mass collected in the substrate was in the range of 2–5 ng.

From the images, geometrical measurements were extracted for ‘each particle’ using the software GENESIS. For each particle, it obtains an image by SEM as well as the mean elemental composition by EDX spectra. Therefore it can also distinguish mixtures of compounds (e.g. sodium chloride with kaolinite), which would not be reliably distinguishable for a single sample by a bulk method. Unless stated otherwise, all diameters used in this manuscript are as given in the projected area equivalent diameter (Dp=√(4*B*/*π*), *B* is the area of the particle), which is close to the aerodynamic diameter for dust particles [30]. A commonly adopted shape descriptor AR obtained from SEM analysis is used. The parameter description of AR as given by the GENESIS and AZTEC software manual is the ratio of the major to the minor axis of the elliptical fit on the projected area [30,31]. When the major axis is *L*, the projected length of an area equivalent sphere of an aspherical particle and the minor axis is the projected area equivalent diameter (Dp) the calculation of AR becomes, *πL*^2^/4*B* [30]. This indicates the shape of the feature with a value of 1 for a circle and higher values for irregular and elongated features [31].

The information on the chemistry of each particle consisting of the major elements F, Na, Mg, Al, Si, P, S, Cl, K, Ca, Ti, V, Cr, Mn, Fe, Zn and Pb is derived from EDX and given as normalized atomic percentages. As the substrate is composed of carbon, information on the elements C and O is discarded while quantifying the particle composition. Owing to the high error, N was not considered. More information on the instrument and analysis can be found in [30,31]. For more information on the particle chemistry, samples collected at various altitudes throughout the measurement period were analysed by electron microscopy for the size range 0.5–3.0 µm and classified into 12 chemical classes. The major dust components were derived by keeping each particle’s Al/Si ratios as the main constraint and the variation of other elements such as Ca, Fe, Mg, K and Na within the aluminosilicates as the second constraint [31]. The main classification schemes used in this study are also given as electronic supplementary material §2.

### 2.3. Auxiliary data

For the number size distribution (NSD) data at SAL conditions, a combination of a Condensation Particle Counter (CPC), Grimm SkyOPC (Optical Particle Counter, 0.25–3.0 µm, uncertainty 5%) and a Cloud and Aerosol Spectrometer—CAS-DPOL (up to 100 µm with uncertainty 10%) was used to derive aerosol particle NSD. The retrieval methods, uncertainty analysis and data quality are detailed in [32]. We used the median NSDs of all SAL segments pertaining to each sampling period (in §5), and the median and s.d. of the total number density of particles (figure 1*e,f*) (in particles cm^3^) for these samples are provided.

Airmass back trajectories reaching the sampling points (sampling points between 2 and 6 km in figure 1*a,b*) were calculated and plotted using the three-dimensional model FLEXTRA (Flexible trajectories) [33]. FLEXTRA is a Lagrangian atmospheric trajectory model used to compute trajectories from gridded meteorological fields of the numerical weather prediction model by the European Centre for Medium-Range Weather Forecasts (ECMWFs). Trajectories are obtained at a 1-h resolution based on calculations using ERA 5 reanalysis data with a spatial resolution of 0.25°.

### 2.4. Calculation of Stoke’s form factor and the settling velocity for aspherical particles

In Stoke’s regime, the gravitational settling of spherical dust particles is calculated after [26,34] as VT_sp_ (cm s^−1^),


(2.1) $$VT_{sp}=\left(g\rho_{p}/18\;\eta \right)\times Dp^{2}\times Cc,$$

where *ρ*_p_ is the density of dust particles, *η* ≈ 1.81 × 10^−5^ kg ms^−1^, *g* is the gravitational constant and Dp is the PAD [35,36]. The slip correction for a particle (Cc) at standard conditions is given as 1+((2.52*λ*)/*D*) [34], where *λ* is the mean free path.

Meanwhile with the non-sphericity of the particle, its drag coefficient increases as compared with its spherical counterpart. So to calculate the terminal velocity of a falling aspherical particle ( [37]) introduced a shape descriptor (*F*_s_= *d*^3^_eq_ / (*L*^2.3^×Dp ^0.7^)_,_ Stoke’s form factor) which can provide tridimensional properties of the particles. In microscope measurements, these three dimensions are the major axis or the longest length of the particle projection generally given as *L*, the minor axis of an equivalent sphere given as *D*_p_ (PAD) and the equivalent diameter *d*_eq_. The additional parameter *d*_eq_ given in [37] is defined as the particle spherical equivalent diameter, or in this study, the volume equivalent diameter, the diameter of a sphere having the same volume as that of an irregular particle.

*F*_s_ value 1 indicates a sphere and decreases with shape deviations.

Hence for each particle, the terminal velocity (VT_as_) is calculated from Stoke’s drag correction factor *γ,*


(2.2) $$\frac{1}{\gamma }\text{=}\frac{{\text{VT}}_{\text{sp}}}{{\text{VT}}_{\text{as}}}\text{=1/2}\left(F_{\text{s}}^{\text{1/3}}\text{+}\frac{\text{1}}{F_{\text{s}}^{\text{1/3}}}\right).$$

Finally, the reduction in terminal velocity for each particle is calculated as (1− (VT_as_ / VT_sp_)) and the enhancement in gravitational settling lifetime ((VT_sp_ / VT_as_) −1) [25].

### 2.5. Fe oxides, Fe index and optical properties calculations

For the representation of Fe content in dust, we used two parameters, (i) the total iron oxide percentage (Fe^w^_oxides_) which exists either as pure oxyhydroxides (hematite or goethite) or in the crystal lattice of a mineral particle [38] and (ii) the total Fe index defined as the atomic ratio of the concentration of Fe and the sum of the concentrations of all the elements quantified [10]. Fe^w^_oxides_ is calculated as,


(2.3) $${\text{Fe}}_{\text{oxides}}^{\text{w}}\text{=}\left(\frac{{\text{MC}}_{\text{Feox}\%}}{{\text{MC}}_{\text{Fe}\%}}\right)\times \;\frac{m_{\text{Fe}}}{m_{\text{dry}}}\times \;\text{100},$$

where MC _Feox%_ and MC_Fe%_ are the mass fractions of iron oxides and the fractional mass of elemental iron to the total dust mass concentration, respectively. These values were taken from table 3 in [20] for various source regions in the Sahara. *m*_Fe_ is the estimated Fe mass in a single-particle from our measurements and *m*_dry_ is the dry mass of the particle.

The Fe index is estimated as,

(2.4) ,$$\left|X_{i}\right|=\frac{X_{i}}{\sum X}$$

with |*X*_i_| element concentration index of a particular element with arbitrary index, here Fe index and Σ⟨*X*⟩ are the sum of all considered elements (Na, Mg, Al, Si, P, S, Cl, K, Ca, Ti, Cr, Mn, Fe and Co) [30,31].

The spectral (0.37–0.95 µm) optical properties for dust used in this study are empirically estimated from the linear fit relation of optical properties, *k* (imaginary part of dust refractive index) and SSA with the total iron oxide concentration in dust (Fe^w^_oxides_). This parametrization is derived by [20] and is given as,

(2.5) $$k,\text{SSA}=a{\text{Fe}}_{\text{oxides}}^{\text{w}}+b,$$

Fe^w^_oxides_ are derived for each particle using (2.3). Coefficients *a* and *b* are taken from table 6 of [20] for *k* and SSA. For mass absorption efficiency (MAE) calculation, the same linear fit equation is used with the values for *a* and *b* derived from figure 4 of [39].

### 2.6. Statistics analysis

The statistical analysis was done in this work to determine the variability of median data from region-wise grouped samples. For this, bootstrapping of data was done for parameters AR, Fe oxide percentage and optical parameters k, SSA and MAE. The boot package in R programming (R version 4.3.3) was used to replicate 10 000 particles. A function is called which returned the median of these data and their confidence intervals between 2.5% and 97.5%.

For independent sample *t*-tests or hypothesis tests, the Wilcox *t*‐test is done to see if the difference between the two regions is significant.

## 3. Results

### 3.1. Overall particle composition in CV and the Caribbean during SALTRACE

In this section we compare samples collected on the eastern tropical Atlantic Ocean—termed ‘Cape Verde (CV)’—with those collected on the Western side of the Ocean—termed ‘Caribbean’. Samples on the Western side contain measurements near Barbados, Antigua and Puerto Rico (cf. figure 1*c,d*).

The major dust classes found [10,31] are clay minerals, other silicates, feldspar, Ca-rich and Fe-rich oxides and hydroxides. Note that, these denominations are descriptive, but do not necessarily identify a mineral. The non-dust part includes sea-salts, sulphates and their mixtures (figure 1*e*) with their typical composition ratios as given in previous studies [30] and the references therein. Figure 1*e,f* shows the overall composition of the particles measured from CV and the Caribbean.

**Figure 2.**
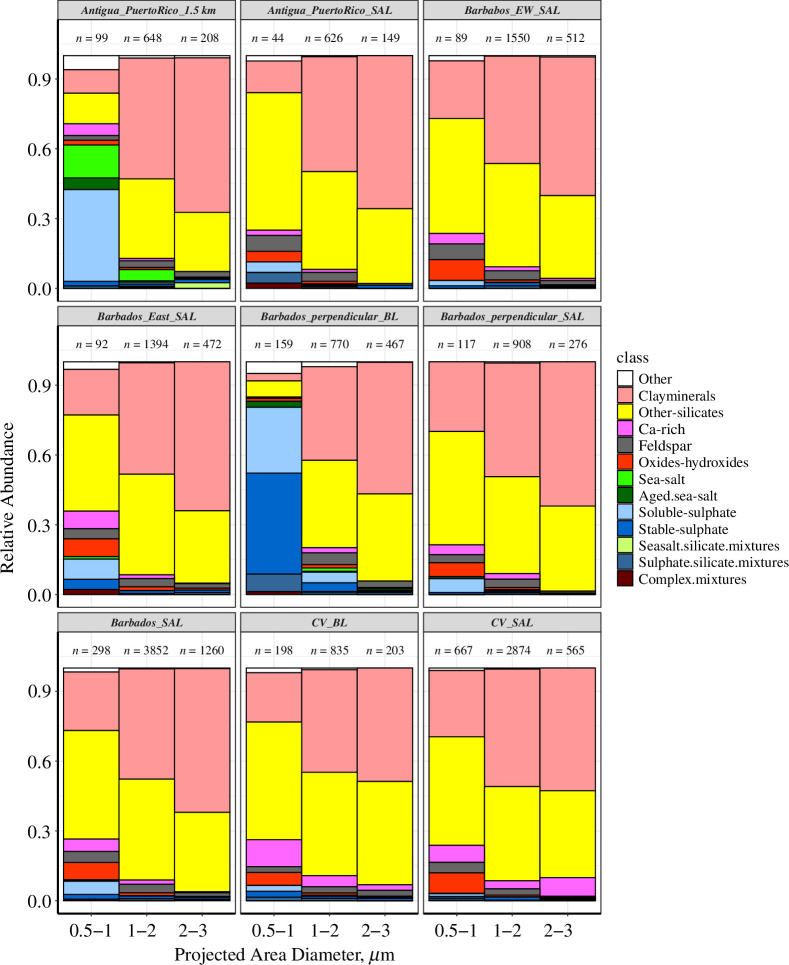
Variability in composition: the variability in size-resolved chemical abundance in the BL and SAL of various regions specified by table 1 (numbers above each stack bar are the total number of particles present in each PAD range).

Over the CV in terms of composition, the SAL existed as a homogeneous layer from 2 to 6 km for the measuring period 11–17 June 2013 during which high dust loading conditions prevailed on 11, 12 and 17 June with AOD values ranging from 0.4 to 0.6 [22] with the highest on 17 June 2013. The particle number concentration (for size range 0.5–8.0 µm) for the entire measurement period in CV varied from 20 to 40 particles cm^−3^ STP. The relative abundance of aerosol components also revealed a consistent composition over the SAL in the CV region except for a single sample with higher Ca-rich particle content collected at 5.5 km on 11 June 2013. A total of ~5800 particles were studied from the CV region which showed the dominance of dust with the major group (42–47%) being clay minerals with a high Al/Si ratio followed by other silicate-like (with low Al/Si ratio) particles (38–42%). Feldspars and iron oxides/hydroxides which influence climate due to their ice nucleation [40] and light absorption properties [38], respectively, were minor groups (feldspar—2–3% and oxides/hydroxides with 1–2%) with dominance in the SAL (region and altitude definitions in electronic supplementary file §1). Figure 2 shows the number abundance of various particle classes from the Eastern Atlantic (CV) and the Caribbean (to show variability throughout the campaign) using single-particle classification. While the dominance of dust was observed over CV from the BL up to 6 km [41] altitude constituting >90% of total aerosol particles, the sea-salts, sulphates and mixtures were less than 10%. After the transatlantic transport of over 4000 km, the overall SAL composition was dust-dominant with certain changes observed in dust mineralogy which is detailed in §3.2. Relative abundance of ~7000 particles measured over Barbados and nearby islands of the Caribbean were used to delineate these changes. Hence the overall composition of the Caribbean during the measurement period (June/July) can be treated as dust-dominant from 2 to ~4 km altitude.

### 3.2. Dust mineralogy from CV and Barbados

**Figure 3.**
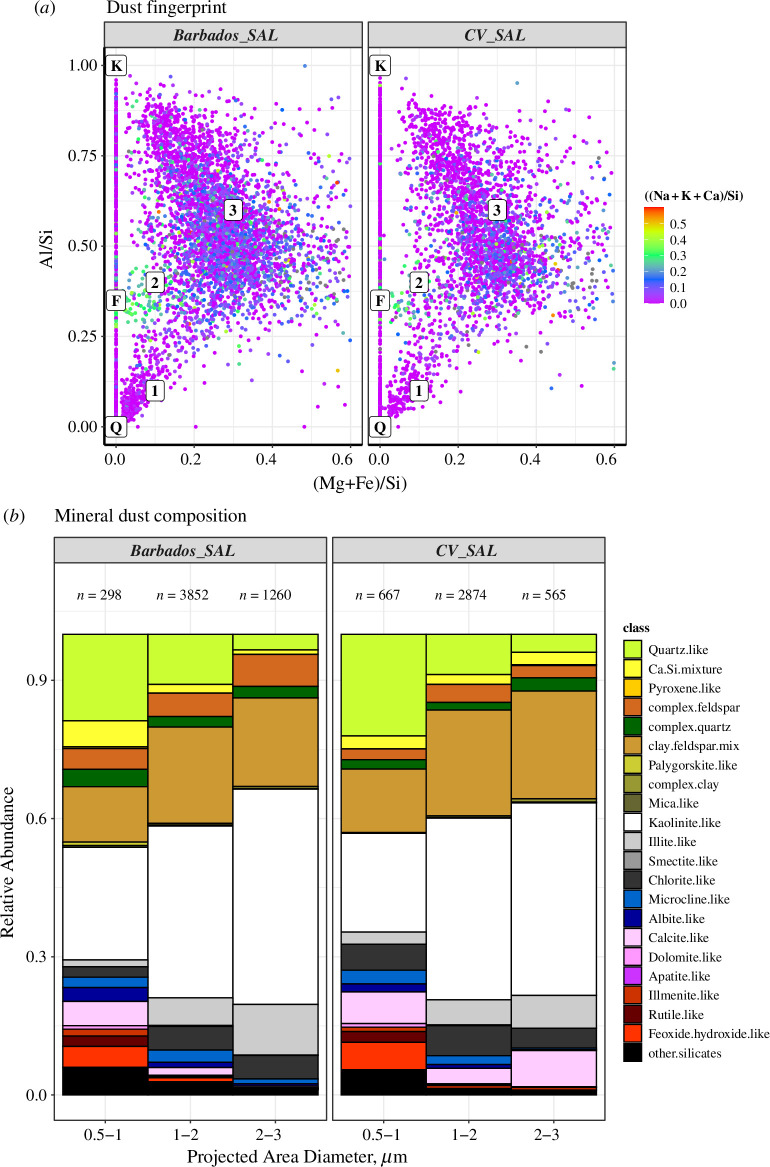
The dust fingerprint over the two study locations along with the major dust groups in the SAL. (*a*) The dust fingerprint of the major silicate dust classes; clay minerals, feldspar and other silicates in the SAL over CV and Barbados. The elemental ratio for the pure quartz (*Q*), K-feldspar (*F*) and the clay mineral kaolinite (*K*) as measured from X-ray Diffraction during the SAMUM campaign [42] is given as symbols along the *Y* axis at (Mg+Fe)/Si value 0, and the numbers 1, 2 and 3 indicates the three clusters/groups observed; (*b*) The derived mineralogical composition of the dust as observed in the SAL before and after transport.

Samples collected during the first half of the campaign constituting a high dust loading period (11–22 June 2013) are only utilized here in figure 3*a,b*, as well as in the sections describing changes with transport. This is done to ensure that the transported dust mineralogy from the Eastern Atlantic (near CV Islands) and Barbados is consecutively represented. The SAL from these two regions are from now on referred to as Barbados SAL and CVSAL, throughout the article. Rigorous sampling was done in Barbados to guarantee an accurate representation of dust composition.

The dominant dust particles in all the samples were mainly rich in silicate-aggregates and had inhomogeneous mineralogical composition. As explained earlier and observed from various reported measurements from Africa, the elemental ratios can be used as a fingerprint of sources as well as to identify the mineralogical composition of each particle [12,43]. Here, also, a dust fingerprint is used to identify the major mineral types using three elemental ratios; first, the (Mg+Fe)/Si ratio which is an indicator of clay mineral aggregates, and feldspars are low in these elements [27,44]. Second, the Al/Si ratio is the ratio of two main components which is the tracer for crustal particles and significantly varies between different minerals. Finally, the (Na+K+ Ca)/Si ratio can be used to separate clay mineral and feldspar groups due to the higher values of the ratio for the latter. These ratios were used for all particles (5800 from CV and 7000 from Barbados) classified in the main classes as clay minerals-like, feldspar-like and other silicates-like. The chemical fingerprint for SAL in CV and Barbados (figure 3*a*) gives a similar clustering to that observed in 2007 over Praia and CV during the SAMUM campaigns [42]. Cluster 1 existing close to the quartz elemental ratio (*Q* in the figure) is from low-impurity Si-dominated particles, Cluster 2 (green colour) rich in (Na+K+ Ca)/Si identified as feldspar-like particles and the third cluster spreads out from the centre of the figure with varying values in the three axes aims towards a flexible mixed clay/silicates group classification.

Apart from the coarse classification into different dust and non-dust components, the dust component was classified into 22 groups, which are defined by certain elemental ratios resembling different minerals. The full scheme of classification is given in [30] also in the electronic supplementary file §2.

The dust particles grouped into 22 mineral groups were segregated according to their diameter, and their normalized relative abundances in each size range are shown in figure 3*b*. This mineralogical composition of SAL before and after transport shows almost similar number abundance in various dust groups with a dominance of silicates and clay mineral-like (kaolinite, illite, smectite, chlorite) particles. Pure and low-impurity ‘quartz-like’ minerals constituted 5–20% at a size range of 0.5–3 µm decreasing towards higher size. Other silicates with varying Al, Mg, K and Ca contents (Ca/Si mixture, complex quartz, complex feldspar and clay-feldspar mix) were 20–30% with increasing abundance in coarse mode. Clay mineral types, for example kaolinite-like particles, were the most abundant with 20–60% in the coarser mode. As reported from earlier single-particle analysis for dust collected from Africa as well as after long-range transport, the clay minerals-like accounted for 60–70% of the dust [9,42,45]. The mean Si/Al ratio for each sample was in the range of ~2 with less variability which is comparable to the value reported from the source regions of northern Mali and southern Algeria [12] (Adrar–Hoggar–Air Mountain region).

**Figure 4.**
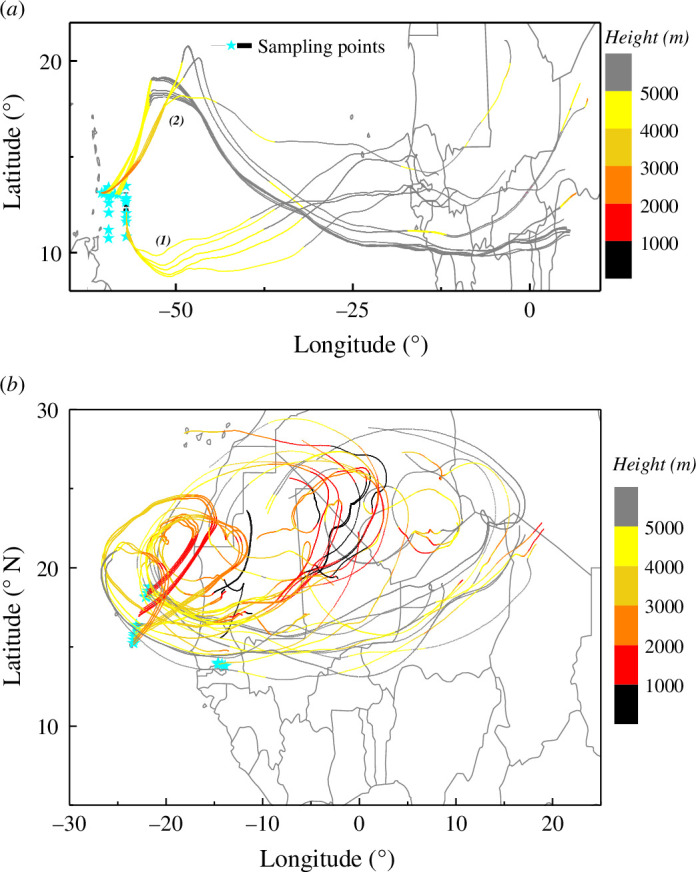
Air mass back trajectories: seven-day airmass back trajectories calculated by the FLEXTRA model for the airmasses reaching various sampling heights (only for SAL) for (*a*) Caribbean Islands and (*b*) CV and Eastern Atlantic Ocean (colour codes—altitudes).

The FLEXTRA back-trajectory calculations show the airmass origin from the South and Northwest Sahara (figure 4*a,b*). The seven-day airmass (figure 4*b*) arriving at the Eastern Atlantic sampling points (>2 km altitude) from 11 to 17 June 2013 originated from the northern part of Africa and followed a counter-clockwise path before reaching the sampling location. The dust source activity maps (fig. 5 of [22]) showed a dust active location between 15°N and 20°N throughout the measurement period (10 June–15 July 2013), with dust source hotspots in the northern part of the Sahara and Maghreb regions [22,46].

**Figure 5.**
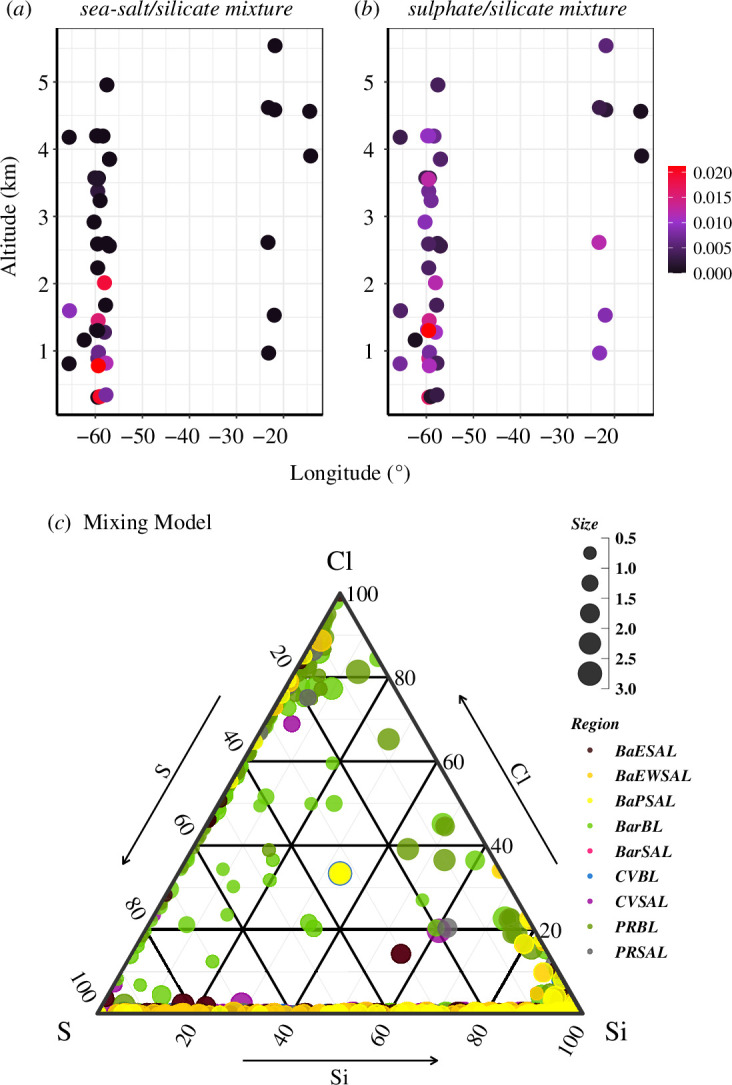
Main particle mixtures observed over the two locations: number abundance of particles in (*a*) sea-salt/silicate mixtures and (*b*) sulphate/silicate mixture class distributed over CV and Caribbean with various altitudes. (*c*) Particle mixing model between Si, S and Cl with Size, PAD (µm) for nine study regions given in table 1.

The air masses reaching the Western Atlantic during 20–22 June 2013 (Cluster 1 as shown in figure 4*a*) originated from the well-mixed SAL (>4 km) between 12°N and 20°N latitude of Northern Africa. The AOD maps from 17 to 22 June 2013 from COSMO–MUSCAT simulations (fig. 8 in [22]) also showed this transatlantic dust transport with the same dust regions, so we can conclude that the source situation was relatively stable. The Western Atlantic sampling location and time were chosen in a way that most measurements had back trajectories (cluster 1, >4 km) crossing the previous sampling region over the Eastern Atlantic. Due to the well-mixed nature of SAL and the stable dust source situation prevalent throughout the measurement period, we can assume that the changes observed are mainly due to transport.

The main difference in mineralogy for the two SALs is the reduction of the calcite-like particles (Ca-rich) for the >1 µm range in Barbados SAL. A 3% reduction in the chlorite-like clay mineral can also be seen in the <1 µm range. The presence of Fe- and Ti-rich particles (hematite/goethite, rutile and ilmenite) as ‘oxides and hydroxides-like’ is of great importance because of their absorption in the shortwave spectrum. The highest percentage of the oxides/hydroxides was observed for particles <1 µm, which accounted for 10% of the total dust abundance with hematite at 4–6%. This is also in agreement with the mass fraction of Fe measured from main dust sources in Africa [47].

### 3.3. Particle mixing state and changes with transport

The particle classification, given in §3.1, indicates dust dominance in both locations with a smaller contribution from sea-salt and sulphates. As we look into the number abundance of total particles, the relative abundance of sea-salt/silicate and sulphate/silicate mixtures was low (<1%) (figure 5*a,b*). With contribution being 0–0.5% over CV and 0.1–1% over Barbados within the PAD range of 0.5–2.5 µm for sea-salt/silicate and sulphate/silicate mixtures, respectively. Figure 5*a,b* shows the altitude-to-longitude variations in the abundance of these mixtures averaged over a sample. It is evident that an internal mixture of sea-salt and silicate is absent (black solid circles) in the higher altitudes of CV which is in agreement with the ground measurements carried out from Praia in 2003 [27]. This was attributed to the transport of dust inside the SAL at mid-range relative humidities (RH varied between 20% and 50% over the sampling points throughout CV) which was ineffectual for the formation of dust/salt mixtures. In addition, the number of sea-salt and aged sea-salt particles was practically none (<0.01%) at these measurement points (altitudes) which deters any possibility of dust/salt mixing.

Over the Caribbean, during the first half of the measurements focused in Barbados, and the dominant dust period, at higher altitudes the contribution of sea-salt as well as sea-salt/silicate mixtures was less than 0.5%. The numbers of sea-salt particles and their mixtures started to pick up in the BL of Puerto Rico and East of Antigua (figure 2, ~10%) and also in the later stage of Barbados measurement after the passage of tropical storm Chantal (9 July 2013). Compared with the sea-salt/silicate mixture, a significant internal mixture of sulphate and mineral dust (1–2%) was observed at both locations. Figure 5*b* indicates the presence of mixtures with mineral dust and sulphates at higher altitudes (SAL) in the Caribbean and at lower altitudes for CV. The ground measurements conducted over Ragged Point (Barbados [30]) during the same measurement period (June/July 2013) indicated approximately 1% of silicate/sulphate mixtures contribute to the total deposited particles [30].

#### 3.3.1. The three-component (ternary) mixing model

A ternary mixing model (figure 5*c*) with the element indices of the three main components Si, S and Cl (element indices: ratio of the specific element to the sum of all elements from equation (2.3)) was plotted for the particles with |Si+S+ Cl|>0.4 [27]. A table of acronyms of the regions is given in table 1, and these regions are also marked in figure 1*b*. The total number of particles analysed with a PAD <3 µm was studied here, which shows a basic binary mixture of dust, sea-salt and sulphates. The edge with increasing Cl gives the binary mixing of dust with sea-salt, which only exists for particles >2 µm, and is unique for the BL of Barbados, Puerto Rico and Antigua regions (light and dark green points). Except for these three regions, a silicate/sea-salt mixture was absent. The increasing S edge shows the ageing of Cl particles which were also limited to the BL regions of PR and Barbados. The silicate/sulphate mixture was the main internal mixture found inside the SAL, at all sites.

The SAL measured perpendicular to the main dust outflow along 59.2°W (BaPSAL (as in figure 1*b*), light yellow) under high dust load conditions had most particles in the pure silicate region with a changing silicate/sulphate mixing of 0–40% (low S/Si ratio). The SAL measured in the east–west direction of Barbados under a low dust load (dark yellow) had a higher S/Si ratio. We compared these results with previous measurements conducted in CV during winter (SAMUM campaign [27]). SAMUM values indicated that the mixing of dust with sulphates was dominant in the upper dust layers and dust/sea-salt mixtures were dominant at ground level as well as <500 m altitudes. The reason for this during the winter can be explained by the airmasses transported near and sometimes within the marine BL [48] promoting the mixing of dust with soluble species. By analysing these two measurements, we can conclude that the dust layer transported to the Atlantic (East and West) during summer months is dominated by internal mixtures of dust and dust/sulphates (excluding the organic content), while the dust/sea-salt mixing depends mainly on the concentration of sea-salt and humidity conditions in the airmass.

#### 3.3.2. Species mixing in the Caribbean

**Figure 6.**
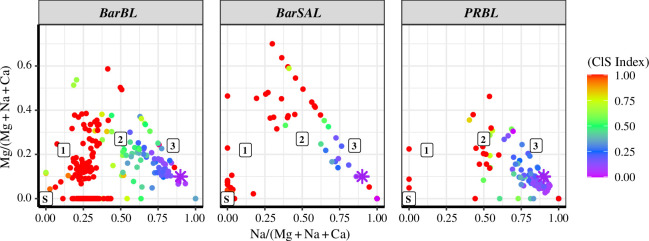
Soluble species and sea-salt ageing: relation between the major inorganic soluble components with sea-salt and sulphates for particles from Barbados boundary layer and Barbados SAL (BarBL and BarSAL) and the boundary layer near Puerto Rico (PRBL). The colour index gives the ClS index, i.e. the ratio S/(S+Cl), lower values indicate sea-salt-rich particles (denoted as a dark purple asterisk in the figure) and values increase with reaction with sulphates.

To better understand species mixing with pure dust in the Caribbean, the ratio of Mg/(Mg+Na+ Ca) versus Na/(Mg+Na+ Ca) was plotted along with the Cl–S index (S/(Cl+S)) for all particles classified as sea-salts, sulphates and dust mixtures. Figure 6 shows the differences in soluble species measured over the Caribbean. As these particles were negligible (<1%) in the Eastern Atlantic, this analysis was not done for CV samples. The boundary layer in Barbados (BarBL) shows two distinct sources. The first cluster with a high Cl–S index and low Mg-to-Na values indicates the presence of gypsum-like (CaS) particles from local emissions or transported dust. CaS can have a marine, geogenic or anthropogenic origin, with dominant formation mechanisms being fractional crystallization of seawater, cloud processing, formation of gypsum with needle structure, the reaction of calcites with atmospheric SO_2_ or H_2_SO_4_, as reported by previous studies [49–52]. A low Cl–S index cluster shows sea-salt and sea-salt ageing. Pure sulphates (e.g. (NH_4_)_2_SO_4_ or gypsum) are marked as S on the left edge of the figure. While cluster 1 indicates high ClS index (red dots) mixtures, or particles with very low Cl content with major elements Na, Mg and SO_4_ (such as Na_2_ Mg (SO_4_)_2_), cluster 2 with mid-range ClS index is a mixture of Na, Mg, Cl and S. Cluster 3 are pure sea-salt like particles with low depletion by sulphate reactions. The pure sea-salt ratio is marked with a purple asterisk in the figure.

The BL near Antigua and Puerto Rico (PRBL in figure 6) has the major soluble species as sea-salt and aged sea-salt with an absence of pure sulphate particles (such as gypsum) unlike what was observed in Barbados. While the Barbados SAL (BarSAL) was low in soluble species, a cluster of pure sulphates (mostly transported gypsum) was observed.

### 3.4. Morphological variations inside SAL

To allow for a more direct comparison of fresh and transported SAL dust in this section, we compare the Eastern Atlantic SAL samples with the Barbados SAL samples, the same as used in §3.2.

#### 3.4.1. Morphology changes of dust types inside SAL

The morphology of the dust particles in the SAL and their changes during transport were investigated here. The dependence of shape on particle diameter has been reported earlier in various studies [31,32,53,54] for short- and long-range transported dust pointing towards the preferential settling of particles with a diameter >4 µm. However, in this study, we focus only on the PAD range 0.5–3 µm which is the more optically active size range in the visible and near-infrared spectra. For a total of 13 000 particles in SAL from CV and Barbados, the size dependence of shape was studied for PAD, 0.5–3.0 µm and is given in table 2.

**Table 2.** Distribution of AR with the PAD for the SAL in Eastern Atlantic and Barbados during high dust loading conditions. The median from bootstrapped data and the central confidence interval (95%) are shown.

region	PAD	**AR** (bootstrapped median and central 95% confidence interval (CCI))	
		**Median**	**CCI**
CV_SAL	0.5–1.0	1.52	1.49–1.56
	1.0–1.5	1.56	1.55–1.58
	1.5–2.0	1.61	1.58–1.64
	2.0–2.5	1.67	1.61–1.73
	2.5–3.0	1.63	1.52–1.72
Barbados_SAL	0.5–1.0	1.49	1.46–1.52
	1.0–1.5	1.51	1.50–1.52
	1.5–2.0	1.58	1.56–1.59
	2.0–2.5	1.66	1.64–1.70
	2.5–3.0	1.72	1.66–1.76

The median AR in CVSAL increases with an increase in particle diameter (0.5–2.5 µm), with values ranging from 1.52 to 1.67, and a decrease is observed between 2.5 and 3.0 µm diameter particles. The central confidence interval at 95% for the bootstrapped median of AR at various size ranges is provided in table 2. A similar increase in median AR with particle diameter is seen for bootstrapped replicates from the Barbados SAL. We also observe a 1–3% decrease in AR after E–W transport in the Barbados SAL for particles with PAD < 2 µm. Due to the narrow size range of particles considered here, this decrease in AR after transport cannot be taken as absolute. Further studies are required to find the change in shape in the higher particle diameter spectrum. A statistical significance test with the R program (Wilcow’s *t*‐test) was carried out for the AR of particles measured from the two SALs. The test gives a *p*‐value > 0.05, indicating a null hypothesis and hence the difference in mean between the two groups is statistically not significant.

To investigate the variation of particle median AR of two dust minerals (silicate-like and clay mineral-like) with altitude inside the SAL, the top and bottom 2 km are considered as upper and lower SAL in the Eastern Atlantic CV (the upper SAL is taken as 4–6 km and below 4 km, the lower SAL), and the top and bottom 1 km in the Caribbean (4–3 km as upper SAL and 3–2 km as lower SAL). Special care was taken in characterizing the samples as lower SAL over the Caribbean because of the convective marine BL and cloud top which varied from 0.5 to 2 km [46]. The dust layer structure from lidar measurements for the measurement period is given in fig. 6 by [22], and we have adapted the same structure in this work [55].

**Figure 7.**
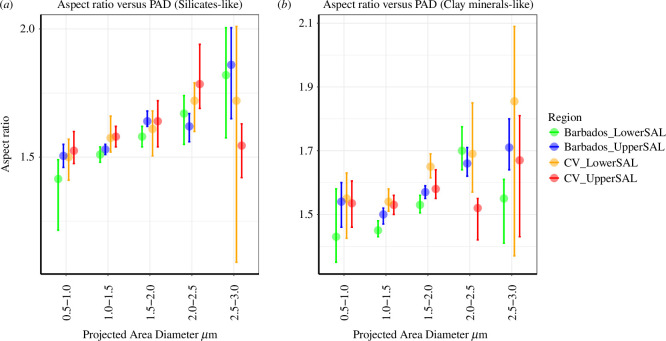
AR versus Particle Diameter: the median AR of (*a*) silicates and (*b*) clay mineral-like particles in the SAL layers of Eastern Atlantic (CV) and Northern Atlantic (Barbados) along with the central confidence interval at 95% for the bootstrapped median (as error bars).

The median AR of two dominant mineral types, clay minerals (Al/Si > 0.5) and other silicate-like minerals (Al/Si < 0.2) were studied for different PAD ranges. The statistics of 10 000 bootstrapped replicates used to get the median and confidence intervals of AR in the SAL layers are given in figure 7 and table 3. Two points can be deduced from this: first, an increase in AR or asphericity was observed when moving towards a higher size (up to 2 µm in upper SAL and 2.5 µm in lower SAL) for both minerals. Secondly in both layers after transport, we observe a small reduction in AR for both mineral types (<1%) in the particle diameter range of 0.5–2.0 µm. However this changes for PAD > 2.0 µm in clay minerals, where an increase in AR is observed after transport. For silicates, this increase is seen at PAD 2.5–3.0 µm. As our measurement data are limited to 3 µm diameter it cannot be deduced if this increase is seen throughout the coarse/giant mode. In real-time transport, the importance of coarse-mode aerosols has to be considered. Overall, during the transatlantic transport of dust, we see a dependency on AR with the PAD 0.5–3.0 µm, and a change in median AR in the upper SAL for the major transported mineral particle types in various size ranges.

**Table 3.** Distribution of AR with the PAD for the SAL in Eastern Atlantic and Barbados during high dust loading conditions, for two main dust types silicates and clay minerals. The median from bootstrapped data and the central confidence interval (95%) are shown.

region	PAD (µm)	median AR (bootstrapped-*R* = 10 000)		central confidence interval (95%)	
		silicates	clay minerals	silicates	clay minerals
CV Upper SAL (*n* = 2700)	0.5–1.0	1.53	1.54	1.46–1.60	1.46–1.61
	1.0–1.5	1.58	1.53	1.54–1.62	1.50–1.56
	1.5–2.0	1.64	1.58	1.54–1.72	1.55–1.64
	2.0–2.5	1.79	1.52	1.69–1.94	1.42–1.55
	2.5–3.0	1.55	1.67	1.42–1.63	1.43–1.81
CV Lower SAL (*n* = 1535)	0.5–1.0	1.50	1.55	1.41–1.57	1.43–1.63
	1.0–1.5	1.58	1.54	1.52–1.66	1.51–1.58
	1.5–2.0	1.61	1.65	1.51–1.68	1.62–1.69
	2.0–2.5	1.72	1.69	1.60–1.79	1.57–1.85
	2.5–3.0	1.72	1.86	1.50–2.01	1.37–2.09
Barbados Upper SAL (*n* = 6000)	0.5–1.0	1.51	1.54	1.46–1.55	1.46–1.6
	1.0–1.5	1.53	1.50	1.51–1.55	1.47–1.52
	1.5–2.0	1.64	1.57	1.62–1.68	1.55–1.59
	2.0–2.5	1.62	1.66	1.56–1.67	1.62–1.71
	2.5–3.0	1.86	1.71	1.65–2.01	1.64–1.80
Barbados Lower SAL (*n* = 2530)	0.5–1.0	1.41	1.43	1.22–1.49	1.35–1.58
	1.0–1.5	1.51	1.45	1.48–1.54	1.43–1.48
	1.5–2.0	1.58	1.53	1.54–1.62	1.51–1.56
	2.0–2.5	1.67	1.70	1.55–1.74	1.64–1.76
	2.5–3.0	1.82	1.55	1.56–2.01	1.41–1.61

Single-particle ground measurements performed over North Africa [9], indicating an increase in AR up to ~2.0 µm, and the variability was less distinct with the increase in size. AER-D flight measurements conducted in the Eastern Atlantic during August 2015 reported an AR distribution for up to a diameter of 10.0 µm, the median AR for the particles 0.5–5.0 µm were between 1.35 and 1.44 which increased with particle diameter [17]. A recent study from Morocco during the 2019 measurements [31] also reported the size-resolved AR distribution but with less variability. Henceforth indicating a strong variability in AR-particle diameter distribution for near-source and transported dust particles. But more studies are required to study the actual physical processes involved, by covering a wide particle size distribution for transported dust.

#### 3.4.2. Dust asphericity and terminal velocity changes of minerals inside SAL

Here we attempt to estimate the gravitational settling of *in situ* measured dust particles with the spherical assumption (VT_sp_) and their variation after applying the correction factor for asphericity (*γ*) (equation (2.1) in §2.4). Various correction factors are available in the literature, including the dynamic shape factor and Stoke’s/Newton’s drag corrections which use Reynold’s number (*Re*) along with shape descriptors (e.g. sphericity, circularity, elongation and flatness) to describe the correlations for predicting the drag force of irregular particles [25,56,57]. Because the dust settling inside the SAL occurs in Stoke’s regime, with Reynolds number Re<1, our calculations and correction factor are constrained to Stoke’s drag correction (1/*γ*) explained in §2.4.

The dependency of the gravitational settling velocity on dust asphericity was evaluated for the two major dust mineral types based on a recent parametrization scheme applied and validated for particles in the Stokes regime [25,37]. The asphericity factor for irregular particles, γ is defined as the ratio of the drag coefficient of an irregular particle to that of a sphere with the same volume. The terminal velocity of the non-spherical particles (VT_as_) is calculated from equation (2.1) in §2.4. As the samples were taken at different pressure levels, the corresponding mean-free path and slip correction (Cc) values were considered while calculating the settling velocity for a spherical particle (the sensitivity of Cc with ambient pressure is given in electronic supplementary material §2 figure 1). From the laboratory calculations as in [37], Stoke’s form factor (*F*_s_, the shape descriptor in §2.4) has shown a better correlation with the drag correction (1/*γ*) for ellipsoids and other isometric shapes with a mean error of 2.4% [37] and maximum error of 33.9%.

Our calculations show a reduction of settling velocity by 15–40% in size ranges from 0.5 to 3 µm in the Barbados SAL, enhancing the lifetime of dust up to 20% (table 4) in the upper SAL layer. These reductions in settling velocity values are also comparable to those reported in [25] where a similar calculation approach was used. As compared with the Caribbean, the velocity reduction is lower in the CV SAL, with a maximum of 11% increase in lifetime in the upper CV SAL (table 4). The settling velocity of particles inside SAL increased with size from PAD 0.5 to 3.0 µm while assuming the spherical nature of the particles (Vt_sp_). Approximately 4–9 times increase was seen in VT_sp_ between 0.5 and 3.0 µm PAD. On the other hand, with the addition of the asphericity form factor, the velocity increase with size was reduced. Particles in the upper layer of CV SAL settle with a median velocity VT_as_ of 0.015–0.02 cm s^–1^ for sizes from 2 to 3 µm in diameter, nevertheless, the Caribbean SAL has a further reduced velocity of 0.003–0.004 cm s^–1^. Recent modelling studies have taken into consideration a 40% reduction in settling velocity for coarse and giant mode (up to 100 µm) to represent the airborne dust downwind of the Eastern Atlantic [58].

**Table 4.** Median settling velocities and lifetime enhancement calculated for particles in various size ranges. The data are given for the upper and lower SAL sampled from the two measurement locations.

region	PAD (µm)	VT_sp (cm/s)_	VT_as_ _(cm/s)_	PAD (µm)	lifetime enhancement
Barbados Lower SAL	0.5–1.0	0.0044	0.004		
	1.0–1.5	0.0069	0.0056	0.5–1.5	11.5%
	1.5–2.0	0.0092	0.0066	1.5–3.0	14.3%
	2.0–2.5	0.0128	0.0075		
	2.5–3.0	0.0211	0.0064		
Barbados Upper SAL	0.5–1.0	0.0029	0.0022		
	1.0–1.5	0.0047	0.0034	0.5–1.5	17.8%
	1.5–2.0	0.0063	0.0037	1.5–3.0	20.15%
	2.0–2.5	0.0092	0.004		
	2.5–3.0	0.0134	0.0031		
CV Lower SAL	0.5–1.0	0.0046	0.0041		
	1.0–1.5	0.0069	0.0058	0.5–1.5	9.8%
	1.5–2.0	0.0094	0.0054	1.5–3.0	13.3%
	2.0–2.5	0.0186	0.0107		
	2.5–3.0	0.0346	0.0102		
CV Upper SAL	0.5–1.0	0.0042	0.0038		
	1.0–1.5	0.0099	0.0090	0.5–1.5	6.8%
	1.5–2.0	0.0158	0.0136	1.5–3.0	11.6%
	2.0–2.5	0.0264	0.0192		
	2.5–3.0	0.0401	0.0284		

### 3.5. Iron content and optical properties changes inside the SAL

#### 3.5.1. Iron index and Fe oxide content change with transport

Here, we adopt two indicators to represent the iron content of the single particles analysed, firstly the ‘Fe index’ for all silicate-rich dust particles is calculated from the ratio of Fe to the sum of all other elements (equation (2.4) in §2.5). The second indicator was the percentage Fe oxide weight (Fe^w^_oxides_) for each particle, calculated from the total Fe elemental fraction multiplied by the fractional contribution representing the Saharan source dust obtained from [20] (§2.5).

**Figure 8.**
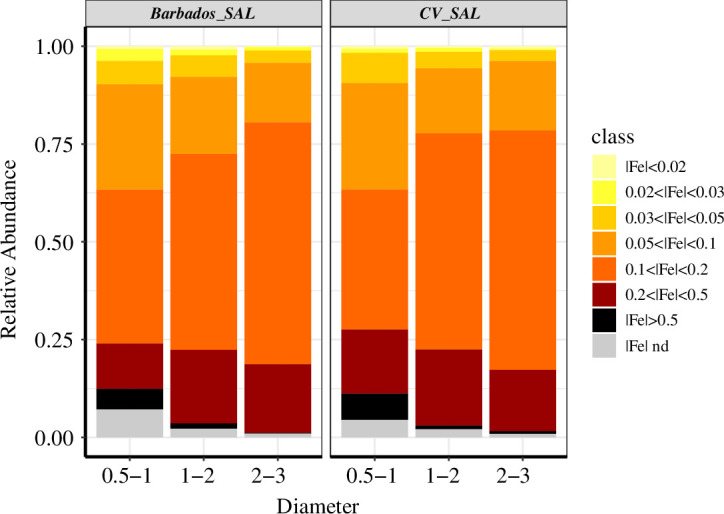
Abundance of Fe in silicate particles: normalized relative abundance of Fe index in each silicate particle for the high dust period in Barbados SAL and CVSAL.

The Fe indices for all silicate-rich particles measured during high dust load conditions over the CV as well as over the Caribbean are shown in figure 8. Particles with |Fe| > 0.5 and 0.2 < |Fe| < 0.5 are mostly Fe oxide/hydroxides (hematite and goethite) or their mixtures with high Al/Si ratio particles (clay-like) [31]. Near-source studies from the Saharan region have shown a steep decrease in the abundance of high Fe-content particles with size (figure 8 from [31]) and after 4 µm no significant amounts were observed. A similar decrease in Fe index is seen here for both locations with increasing particle sizes up to 3 µm. The apparent change seen in the Fe index after transport is for particles of PAD 0.5–1.0 µm with a 27% (0.059–0.042) reduction in |Fe| > 0.5 and ~37% decrease (0.145–0.095) in 0.2 < |Fe| < 0.5.

CV SAL had an abundance of silicate particles with 5% in |Fe| > 0.5 and 14% in 0.2 < |Fe| < 0.5. This observation is similar to the measurement from Tinfou and Praia (5–6%) during the SAMUM campaign [27] where the southerly dust sources of the Sahara are prevalent with more iron-rich dust particles. The reduction in the Fe index after transport to the Caribbean can be explained by the deposition of Fe-rich particles (such as hematite and goethite) or dust particles (<1 µm) which are dominant in iron content. Looking into the variability of Fe composition around Barbados, the indices remained uniform in the EW direction and for samples collected perpendicular to the SAL movement (figure 2 electronic supplement §3).

The second indicator used is the percentage of iron as oxide weight (hereafter referred to as Fe^w^_oxides_) present in ‘each particle’. The bootstrapped statistics of Fe^w^_oxides_ present in the different layers of SAL are given in table 5. The median Fe^w^_oxide_ percentage of total dust remained in the range of 6.0–6.8 for the SAL with a higher value over the CV upper SAL and the lowest in the lower Saharan layer in Barbados (6.15). A decrease in Fe^w^_oxide_ percentage is observed from the upper part of CV SAL to the Barbados SAL in the size range 0.5–1.5 µm indicating the deposition of Fe-rich particles inside the SAL layer during their transport towards the North Atlantic. Although a definite explanation for this reduction in Fe-rich particles after transport cannot be provided at this stage, but few possible reasons can be considered. A possible explanation could be a higher particle density for these Fe-rich particles than previously considered, which could affect their settling velocities.

**Table 5.** Median and 95% confidence level of total percentage Fe oxide content (bootstrapped values), at two size ranges in dust over SAL before and after transport to the Caribbean.

region	median (Feox, %)		central confidence interval (95%).	
	0.5–1.5 µm	1.5–3.0 µm	0.5–1.5 µm	1.5–3.0 µm
Barbados Lower SAL	6.15	6.02	5.85–6.36	5.87–6.21
Barbados Upper SAL	6.58	6.01	6.37–6.74	5.91–6.11
CV Lower SAL	6.76	6.04	6.45–7.06	5.85–6.26
CV Upper SAL	6.84	6.07	6.74–7.08	5.88–6.27

A sensitivity estimation was carried out with a higher density for typical iron oxide particles (5.5 g cm^−3^) to assess the change in terminal velocity. When the density is changed from 2.6 to 5.5 g cm^−3^ [34,59], the terminal velocity increases a maximum of ~55% (for density 5.5 g cm^−3^). As a result, the particles we term as iron-rich probably deposit by 15–55% (when density is increased from 2.7 to 5.5 g cm^−3^) faster in the size range of 0.5–3.0 µm. As the contribution of these particles is higher in the lower size range (<1.5 µm), the reduction is prominent in that particular size range. The second possibility is connected to the particle shape. We observe that the median AR of these submicron particles (<1 µm) ‘rich in iron’ is in the range of <1.45 whose terminal velocity can be higher than their aspherical counterparts. In total, the Fe-rich particles in 0.5–1.5 µm PAD with AR < 1.45 in the Eastern Atlantic constituted ~10% of the total dust while in the Caribbean it was ~8%. Hence the higher density of Fe-rich particles and their low AR could complement their faster deposition in the atmosphere.

#### 3.5.2. Spectral variation of dust absorption parameters and transport changes.

**Figure 9.**
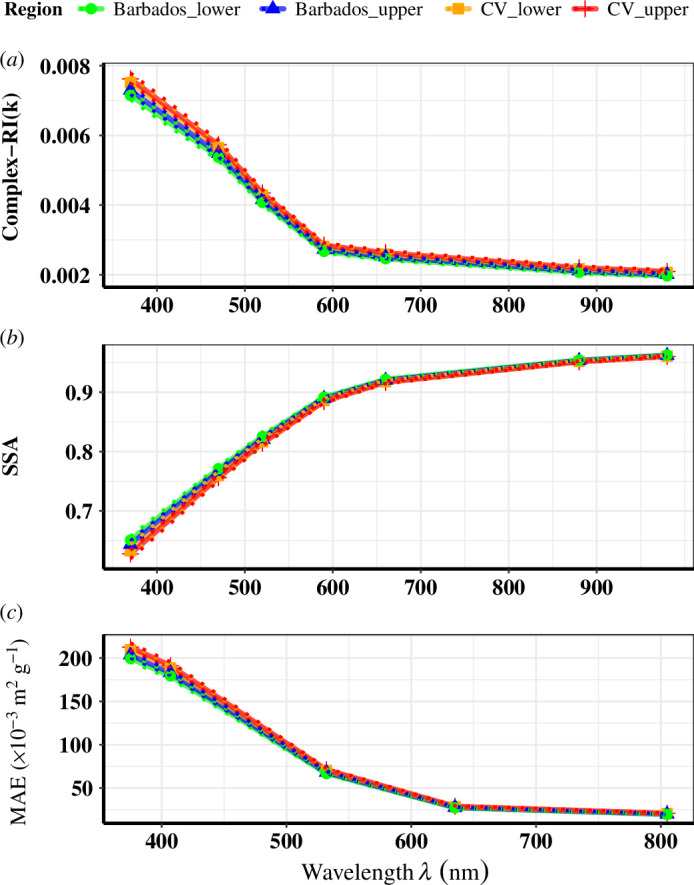
Spectral variations of optical properties for dust particles: median (solid line), 95% (dotted) confidence levels after bootstrapping for (*a*) Imaginary refractive index (*k*), (*b*) SSA and (*c*) mass absorption coefficient in m^2^ g^−1^.

Spectral variation (370–980) nm of the imaginary part of the dust refractive index (*k*) along with the SSA and MAE from a single particle was calculated using equation 2.5 in §2.5. The parameterization of dust absorption as a function of dust mineralogy is presented here. An empirical relationship [39] derived between optical parameters and the total Fe oxides present in the dust as from [20] and [39] are used here. As these relations are dependent on the percentage Fe oxide content (which varies with particle size range) explained in §3.5a, the reported values here apply to transported Saharan dust in the PAD range 0.5–3.0 µm. Figure 9 illustrates the spectral variation (of eight wavelengths from 370 to 980) of these three parameters as measured in the upper and lower regions of the SAL from CV and Barbados in the Caribbean.

In figure 9, the bootstrapped median values from the entire set of analysed samples from each region are given as solid lines with their respective symbols, and their lower and upper confidence values (0.025–0.95) are represented by dotted lines.

A decrease in dust absorption is seen with increasing wavelength, as depicted by the variation of dust imaginary part *k* (figure 9). This is the typical spectral behaviour of mineral dust, as observed from previous measurements [9,60]. The values (upper and lower confidence levels) of the median dust imaginary part of the refractive index (*k*) varied from 0.0075 to 0.0078 at 370 nm and 0.0043–0.0044 at 520 nm in the upper layer of CV SAL (table 6). While in Barbados SAL, the variability was in the range of 0.0072–0.0074 at 370 nm. At 520 nm, the *k* value decreased from 0.0043 in the upper SAL of CV to 0.0041 in the upper SAL Barbados (~4% decrease). The higher absorption values of *k* reported here are reflected in the low SSA values in the upper CV SAL between 0.63 at 370 nm and 0.80 at 520 nm. The SSA changes of dust in upper SAL after transport account for a 2.5% (0.627–0.643) increase at 370 nm and a 1% (0.802–0.809) increase at 520 nm. Varying the SSA of mineral dust over Western Africa by ±5% has shown a drastic shift in regional climate responses based on modelling studies [61], so even these low changes might have a considerable impact.

**Table 6.** Bootstrapped median of imaginary refractive index *k*, SSA, at 520 nm for two size ranges in dust over SAL before and after transport. The 95% confidence intervals are given in brackets.

region (SAL) PAD(µm)	median (*k* × 10^−2^) with 95% CCI		median SSA with 95% CCI	
	0.5–1.5 µm	1.5–3.0 µm	0.5–1.5 µm	1.5–3.0 µm
Barbados Lower	0.42(0.39–0.43)	0.42(0.39–0.4)	0.822(0.816–0.830)	0.825(0.82–0.83)
Barbados Upper	0.44(0.43–0.45)	0.41(0.39–0.41)	0.809(0.804–0.815)	0.826(0.823–0.829)
CV Lower	0.46(0.44–0.48)	0.41(0.4–0.42)	0.804(0.795–0.813)	0.825(0.819–0.830)
CV Upper	0.46(0.46–0.47)	0.41(0.39–0.42)	0.802(0.795–0.804)	0.824(0.18–0.829)
After-storm high dust	0.42(0.40–0.43)	0.42(0.41–0.43)	0.821(0.814–0.827)	0.821(0.815–0.825)

We performed a statistical test (Wilcox test) to see if the difference in *k* values from the upper SAL of two locations (for all wavelengths) was significant. Indeed, the difference in *k* is highly significant (*p* = 5.41 × 10^−8^). The difference was found to be statistically significant even with a robust test having lower sensitivity than a parametric test.

The MAE values were calculated using equations adapted from [39], using the linear relation between Fe oxides (from Sahara) and MAE values for PM2.5 size fraction (§2.5). The median values for spectral variation of MAE in upper CVSAL were ~212 ×10^−3^ m^2^ g^−1^ at 375 nm to 71 × 10^−3^ m^2^ g^−1^ at 532 nm, with 7 times decrease from 375 to 660 nm, while laboratory measurements by [39] reported MAE values from 95 to 132 ×10^–3^ m^2^ g^−1^ for the Northern Sahara and 711 × 10^−3^ m^2^ g^−1^ for the Sahel at 375 nm for PM 2.5 fraction. An intercontinental dust transport study conducted in Puerto Rico (ground station, 66 m asl) in June/July 2012 reported a mean dust MAE as 90 × 10^−3^ m^2^ g^−1^ at 428 nm for PM1 fractions [62].

Aircraft measurements from near-source regions during campaigns DODO1 and 2 (February 2006 and August 2006), DABEX (January 2006) and SHADE (September 2000) reported *k* values (table 7) [13,27,63–65] in the range of 0.004–0.0015 at 550 nm. Flight measurements [66] of single particles using electron microscopy were carried out during the SAMUM 2 winter campaign (February 2008) conducted over Praia/Dakar. The reported spectral *k* values for the study were higher as compared with this study, pertaining to the season with higher soot inclusion and biomass burning particles. Similar measurements from the SAMUM I campaign in May/June 2006 [9] reported comparable k values over Morocco from (6.9 to 0.17) × 10^−3^ for wavelengths of 350–1640 nm. Single-particle dust deposition measurements over ground level conducted [27] during the winter SAMUM campaign in 2008 reported *k* values from 0.009 to 0.011 at 497 nm and 0.0007–0.0009 at 660 nm for particles between 0.5 and 5 µm diameter (table 7). During the same campaign, spectral absorption photometer measurements [67] gave 0.007 at 500 nm and 0.0025 at 650 nm, which were higher than the observed values from single-particle measurements. The main reason for these differences was inferred from the uncertainty in both measurements, with photometer measurements having a higher uncertainty for non-accounting of non-sphericity, and for microscopy measurements, the assumptions going into the calculations of iron oxide percentage.

**Table 7.** Dust refractive indices measured near Africa and on the outflow regions by various *in situ* measurements.

study region	year of study	AR	size range (µm) diameter	*k* (Median)	wavelength	reference
CV	Jan/Feb 2008	1.6–1.7	0.5–1.0	0.0087	532	[27]
			1.0–2.5	0.0064		
			2.5–5.0	0.0074		
CV	June/July 2013	1.55	0.5–1.5	0.0045	520	this study
		1.68	1.5–3.0	0.0041		
Morocco	May/June 2008	1.6–1.8	0.5–10.0	0.0035	530	[9]
South of Dakar	Feb 2006		>1.0	0.001	550	[63]
Mauritania	Aug 2006		>1.0	0.002–0.003	550	[63]
Niamey	Jan 2006			0.004	550	[64]
CV	Sep 2000			0.0015	550	[65]

Although the current method described has its limitations due to the empirical relations used (uncertainty: ±30%), which assumed the spherical shape of particles during the retrieval procedures, a preferably accurate iron percentage calculation for the representative SAL reduces the uncertainty in the final reported values.

#### 3.5.3. Transport changes of dust optical properties with size

**Figure 10.**
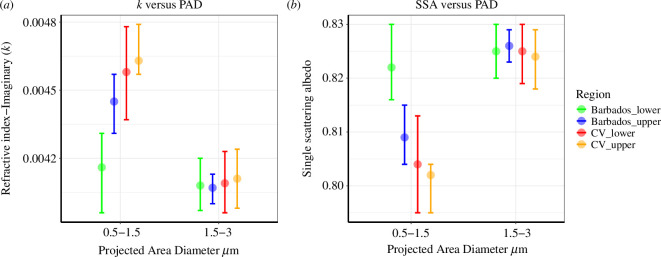
The bootstrapped median for (*a*) dust imaginary refractive index (*k*) and (*b*) dust SSA in the SAL layers of Eastern Atlantic (CV) and Northern Atlantic (Barbados) along with the 95% central confidence level (as error bars).

The optical properties (*k* and SSA) at two size ranges at wavelength 520 nm were studied and are summarized in table 6 and figure 10. The median values and 95% confidence intervals for the four regions are given (bootstrapped with 10 000 replications). For the considered size range (0.5–3.0 µm), *k* values decreased with increasing size indicating a decrease in dust absorption.

The median *k* values at 520 nm for the two size ranges (0.5–1.5 and 1.5–3.0 µm) were in the range of 0.0041–0.0046 in the CVSAL. These values are comparable to the near-source values reported from Africa for size ranges 1–4 µm as (3.5–7) × 10^−3^ [10] at 530 nm. The percentage changes in optical properties inside SAL after the transport as calculated in the previous section (4% in *k* and 1% in SSA §3.5b) were observed inside the upper SAL mainly for the 0.5–1.5 µm size range. While the values for *k* and SSA (at 520 nm) had a smaller change with transport (<1%) in the diameter range of 1.5–3 µm with low variance (indicated by confidence interval). This indicates that the change in the spectral optical properties of dust with transport that we observed here is prominently from the particles with PAD 0.5–1.5 µm. Nevertheless, in the lower SAL, a 9% decrease in *k* values was found in the 0.5–1.5 µm range, while the 1.5–3.0 µm had a 2% decrease.

#### 3.5.4. Dust optical properties in the Caribbean after the passage of tropical storm Chantal

The passage of the tropical storm Chantal (8–10 July 2013) provided a unique opportunity to measure the dust properties before and after the storm. A reduced dust load was observed over Barbados [22,46] before the storm and immediately after the passage, a strong and stable flow of Saharan dust towards the Caribbean was established which was observed as a layer of height up to 4.8 km [46]. The relative chemical composition on 8 July 2013 before the passage of the storm showed a higher percentage of sulphates and sea-salt in the lower size while for size >1 µm the dust dominated. After the storm, dust dominated in all sizes. Hence the optical properties for the after-storm condition during the high dust load period were calculated to determine any variance relative to the previous days. One difference that we can see here is no variability in absorption towards the higher size (table 6), which is opposite to that of the results from the previous days of measurements in the Barbados SAL. This is echoed in the *k*, SSA and MAE values. AOD values reported during this measurement period (10–11 July 2013) were between 0.3 and 0.4 during the Falcon flights [46]. This high dust load event was different from previous cases during the campaign, with an increase in atmospheric humidity over North Africa contributing to deep convective clouds that grow into mesoscale convective systems. This sequentially generated haboobs in West Africa, and thus high dust load conditions originating from the southwest flanks of Hoggar Massif [46]. Thus, even during the same season, the SAL over the Caribbean and its properties depend on various factors including the source regions, and the meteorological conditions favouring the emission and transport conditions.

**Figure 11.**
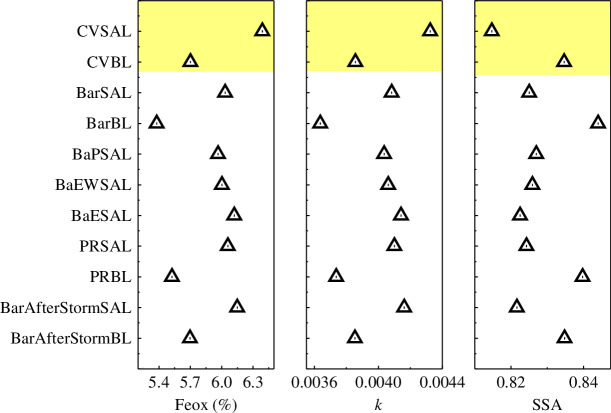
Regional variations of optical properties for dust particles: bootstrapped median values for percentage iron oxides, Imaginary refractive index (*k*) at 520 nm and SSA at 520 nm defined by heights (regions defined in §1 of supplementary file and figure 1*b–d*).

To see the heterogeneity in the absorption characteristics of Caribbean SAL, the *k* and SSA values at 520 nm for the different regions in the Eastern Atlantic and the Caribbean are shown in figure 11. Here, the optical properties of Barbados SAL after the passage of the storm are also shown. The percentage contribution of Fe oxides in the different locations of the Caribbean remained in the range of 6.0–6.1, while in CV SAL it was 6.4. The corresponding *k* and SSA values over the Caribbean were ~0.0041 and ~0.825, respectively. In the BL of both sites, %Fe values were <5.7, and the corresponding SSA values (660 nm) were >0.925. After the storm, the *k* values were ~0.0042 and the corresponding SSA at 0.82.

## 4. Discussion

The main questions we try to address here are, what is the change in composition with altitude before and after the transatlantic transport of dust inside SAL, and do they vary after the passage of storm Chantal? Does gravitational settling play a major role in the SAL and, if so, what is the size and shape dependence? Finally, how are the radiative parameters inside the SAL changed after transport?

### 4.1. Spatial variability of SAL composition

The vertical variability in composition indicated a homogeneous composition between the altitudes 0.5 and 5.5 km over the CV region, suggesting a ‘dusty’ (>90% in number abundance) background above the marine BL along with a well-defined SAL at altitudes 2–5 km. However, the Caribbean SAL retained the percentage dust composition as the near-source region, while in the marine BL, other soluble species increased by 10% (sulphates and sea-salt). A persistent dust layer was observed in the Caribbean during the 1-month campaign period except for a few days during the passage of the tropical storm. The formation and transatlantic transport of this dust layer from Africa normally depends on dust sources active during the period, atmospheric circulation prevailing in the regions of West Africa and their entrainment over the marine BL of the Atlantic region where it exists as the SAL [5,6].

The chemical and mineralogical composition of dust aerosols is often linked to their source regions or precisely to the parent soils even after transport to far distances [41,44]. The most dominant dust particles in the clay fraction (<2 µm) as observed from different source regions of the Sahara and Sahel include clay minerals with high Al/Si ratios, feldspars and Si-rich (quartz-like) particles with lower Al/Si content [12]. Along with the elemental ratios, the mass ratios between the minerals were also preserved in the aerosol form as that of the parent soils. While these ratios can sometimes be used to estimate a coarse source region of the dust [12,68], in the present work, there was not enough variation (~2). A more distinctive approach like using stable isotope analysis was not possible due to the large mass demand for the technique [69]. Therefore potential source regions could only be assessed by meteorological back-trajectory analysis (figure 4*a,b*) [46,55]. The summer season pertaining to this measurement campaign had a few dust storm events with the main active dust sources located near the Adrar–Hoggar–Air Mountain region, Bodele Depression and a few hotspots near West Sahara Mauritania, Senegal and Morocco [46].

Throughout the measurement period, the SAL dust composition (>90%) was uniform over the Caribbean (figure 2), and in the BL between 1 and 2 km, the total composition changed in the lower size range with the presence of more sulphate-containing particles. Dust is eventually deposited once it reaches the atmospheric BL. The dry deposition measurements conducted during the same measurement period at Ragged Point, a ground measurement site located in Barbados Island, reported dust deposition rates in the range of ~100–250 mm^–2^ day^–1^ for particles in the size range 1–4 µm during the first half of the campaign during the high dust load period (§4 figure 3 of the electronic supplementary material).

### 4.2. Dust morphology changes inside SAL

**Figure 12.**
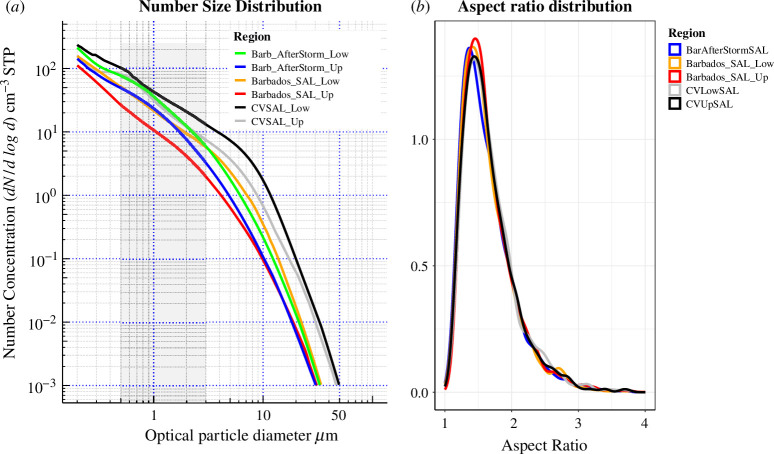
Morphological changes inside SAL: (***a***) the NSD of particles measured from different SAL locations (the shaded area is to highlight the size range 0.5–3.0 µm). (*b*) AR distribution for the different SAL heights at CV and the Caribbean.

The most widely used shape descriptor in microscopic studies, AR is used to describe the change in shape after transport for two size ranges. The NSD and the AR distribution changes during transport are shown in figure 12*a,b*. Overall, we observe a shift in the AR distribution towards the right (increasing AR) during the SAL transport from CV to the Caribbean. The median AR after transport had only a 1% increase in the upper SAL for particles >1.5 µm PAD. This difference in AR cannot be taken as absolute change due to the failure of the statistically significant test.

So for the terminal velocity calculations, a simpler but effective parameter named Stoke’s form factor (*F*_S_) is used [37]. Recent laboratory and modelling studies for dust shape and terminal velocity calculations consider the *F*_S_ as a more robust parameter [25], which uses a tridimensional approach (§2.4) as compared with other shape parameters which are two-dimensional. The corresponding settling velocities calculated are 0.004–0.028 cm s^−1^ for aspherical particles in CVSAL (table 4). For instance, a particle with a diameter of 2 µm inside the SAL with 0.02 cm s^−1^ settling speed or ~17 m day^−1^, descent ~110 m in 6 days. Consequently, a particle at 3.0 µm settling will be faster with ~170 m descent in 6 days. With these current falling speeds (table 4), we calculate for particles from 1.5 to 3.0 µm, a percentage lifetime enhancement of up to 11% for dust in CVSAL and 20% over the Caribbean SAL. This is of course neglecting other factors like diffusion or vertical turbulence during the transport which can affect the actual terminal velocity of particles in the SAL.

For a more quantitative approach, the number distribution of a total number of particles in the size range 0.3–100 µm was measured from inside the Falcon cabin and on the wing stations [32,41]. The number distribution was averaged over the sampling time, and the median was plotted for the respective regions and shown in figure 12*a*. SAL layers in six conditions are considered here with the upper and lower SAL for CV and Barbados. The upper and lower SAL were studied over Barbados after the passage of the storm. The number density distribution showed a higher density in the lower SAL layer for all regions (45–50 cm^−3^). The shaded area between 0.5 and 3 µm is to represent the size range covered in our study. After transport, there was a decrease in the number density across all size ranges (10%). This is expected because, as we move away from the source to a cleaner environment the pollutant (here dust) concentration decreases [70,71]. However, the question that remains is whether this reduction in concentration is dependent on the specific particle properties (morphological and chemical) or the meteorology associated with the transport.

A study investigating the separation of particle sizes inside SAL due to settling was carried out during the same flight measurement [72] for a high dust load event on 22 June 2013. They reported vertical variability of number concentration in two size ranges (5.0–6.0/0.6–1.2 µm) at the top 1 km of SAL. This variability indicates a size-selective deposition inside the SAL on 22 June 2013. However, for similar calculations done after the storm (specifically on 10 and 11 July 2013), the particle number concentration for various size ranges remained uniform within the top 1 km, or size separation was absent. During the same measurement period, two different meteorological conditions showed different vertical variability in the number concentration of particle sizes inside SAL. This suggests that the processes acting towards the size separation can change with the meteorology of the source/destination and the transport pathways. Hence, the gravitational settling of the particles is not always the main contributor to the vertical variability of particles inside the SAL. A similar change in the ratio of small-to-large particles was reported during the PRIDE campaign in Puerto Rico [73,74], although they provided data during the same dry season of June/July 2003 for six dust events with individual characteristics. The asphericity calculations were unaccounted for and hence overestimated the settling velocities for coarse dust particles. They concluded that the vertical distribution of dust over the Caribbean was ‘highly variable’ even when considering a single season, which points towards multiple factors in the act during the transport of SAL.

### 4.3. Change in dust optical properties inside the transported SAL

Changes were observed while examining the optical characteristics of dry dust particles inside the SAL during its transport from the Eastern Atlantic to Barbados in the Caribbean. The *k* values in the upper and lower layer of SAL in CV were analogous, while for the Barbados SAL, the values decreased (for PAD 0.5–1.5 µm) in the lower SAL compared with the upper SAL.

The major changes in the dust absorption (dust imaginary refractive index, *k* and dust SSA) after transport were seen in the PAD range 0.5–1.5 µm. It is crucial to emphasize here that our study exclusively focuses on particles with PAD 0.5–3.0 µm. Furthermore, it is worth noting that the size range (0.5–1.5 µm) has a higher concentration of Fe-rich particles as compared with the particles 1.5–3.0 µm. So, the mean absorption properties can be changed when considering the total absorption of dust in a wide size spectrum.

Considering the overall SAL particles with PAD 0.5–1.5 µm, a reduction in absorption was observed after transport (in upper SAL), with a ~4.2% decrease in the imaginary refractive indices (*k*) at 520 nm with a maximum of 4.5% at 660 nm wavelength. This results in an increase in SSA and hence less absorption due to dust in the Caribbean SAL (2.5% decrease at 370 nm and 1% decrease at 520 nm) as compared with before transport. The changes observed in the optical properties (*k* and SSA) inside SAL after transport have a statistically significant difference (with the Wilcox test) with a *p*‐value << 0.05. While this statistical significance is seen only for the particle with a diameter range of 0.5–1.5 µm, no significant changes were observed for the higher size of 1.5–3.0 µm.

The values of imaginary refractive indices reported here are in the upper range of those reported for soils [20], and consequently, the SSA values are lower. While it cannot be excluded that this might relate to a particular source situation, it should be considered that generally the relative abundance of Fe-rich particles and Fe oxides overall increases with decreasing particle size [10,75]. As a result, it is expected that this study, where mainly particles between 0.5 and 3 µm were investigated, yield a higher Fe-related impact on the optical properties than studies, where a broader size distribution could be analysed. Therefore, one must be cautious when extrapolating these findings to studies where a considerably greater amount of larger particles is expected, and at the same time, only an integral measurement can be done.

## 5. Conclusions

An extensive measurement campaign was conducted in the Eastern Atlantic (CV) and the Caribbean during the summer of June/July 2013, the SALTRACE campaign. Several studies have been reported from this field campaign concerning the active source regions, transport features of dust in the SAL, physical characteristics, optical properties and finally their deposition in the Caribbean [22,30,46,76]. Our study reports the changes in the morphological, chemical and optical properties of ~14 000 single particles measured inside the SAL through multiple flight measurements conducted near the source region (CV) and over the Caribbean after its transport.

Owing to the measurement season, with multiple active source regions in the Sahara/Sahel region, the overall measurement period was dust dominated with >90% of the total aerosol composition constituting clay-like and silicate-like particles in the 0.5–3.0 µm size range. During the second half of the measurement campaign, the variability of composition in the Caribbean was studied around the Barbados, East of Antigua and Puerto Rico islands, and the SAL composition remained consistent with dust dominance. A distinction in composition was seen at lower altitudes (<1.5 km) for particles smaller than 1 µm size. Mixing of particles was rare inside the SAL and most of the sulphate/silicate mixture and sea-salt/silicate mixtures were confined to the lower BL in the Caribbean. Sea-salt/silicate mixtures were scarce (<0.01%) at different altitudes as measured near the Eastern Atlantic (CV) which is concurrent with the measurement period.

To study the overall change in the shape of particles (for sizes 0.5–1.5 and 1.5–3.0 µm), inside the SAL, a shape descriptor AR was used. A variation in AR with size was observed in both locations. While looking into the changes in the AR of particles after transport inside the upper and lower parts of the SAL, the percentage change (<1%) observed was statistically insignificant and hence cannot be taken into account.

Hence, to calculate the settling speed of aspherical particles in the two size ranges, we adopted the shape descriptors and calculations after (Bagheri and Baradonna, 2016) [37]. With the introduction of the asphericity factor, the settling velocity (Vt_as_) was reduced by 25% for the particles measured over CV which gives a lifetime enhancement of up to 11% (PAD 1.5–3.0 µm) in the CV SAL. Over the Caribbean especially in the upper SAL the median particle lifetime enhancement was calculated by up to 20% (PAD 1.5–3.0 µm). This is also seen in higher values of AR in the upper layer of the SAL over the Caribbean (for particles 2–3 µm).

The changes are seen in the optical properties inside the SAL after transport, but are mostly due to the decrease in iron oxide percentage present at different altitudes. The decrease in the percentage of iron oxides (4%) observed in the SAL after transatlantic transport, and thereby the decrease in dust absorption of SAL (2.5% decrease in dust imaginary refractive index at 550 nm) dust was confined to the lower size range (0.5–1.5 µm). The *t*‐test indicated a significant difference in absorption properties between the two locations in this size range. No significant differences were observed in the higher size range (1.5–3.0 µm). One of the reasons for this reduction in transport is the faster deposition of iron oxide particles. Sensitivity analysis for terminal velocity of iron oxide particles by changing the densities 2600–5500 kg m^−3^ showed that these particles settle faster by up to 55 with the increase in particle density. Recent modelling studies have considered differentiating the densities and optical properties between mineral classes [77,78]. These studies reported that this implementation does not change the overall results in terms of aerosol optical depth, surface concentrations and deposition fluxes. However, for future studies on biogeochemical studies, this information on density and refractive indices of differentiated mineral species is important [77].

The systematic uncertainty of our approach for optical properties calculations arises from the ESEM measurements and the parametrization relation used. As a result, we estimate the trueness of the measurement to be in the range of ±40% of the values given, but the precision of measurement, which is relevant for comparing the samples to each other, is in the range of 10%.

The main limitation of our approach is that the calculations for the optical properties do not consider the shape of the particle. If one takes into account that the most absorptive iron-rich particles have a more sphere-like shape than the average dust and therefore scatter less efficiently [79,80], it can be assumed that the SSA is affected more strongly. Using the spherical shape model underestimates the scattering efficiencies by ~4% and most of the recent dust modelling studies favour an ellipsoidal shape model rather than a spherical one [58,80]. As a result, it will need more concurrent studies from both locations focusing on the change in dust mineralogy and its optical properties, with transport, which take into account shape properties and mineralogical properties at the same time.

It is to be taken into account that our measurements are for only a short size spectrum (0.5–3.0 µm) and the optical properties summarized are for the same. The iron oxides have maximum abundance in this range. Our study has not addressed the properties of particles with a diameter >3.0 µm, the size range in which most of the recent modelling studies are interested [19]. Hence for a comprehensive explanation of optical property changes with transport, more advanced studies constituting the whole size spectrum are required in future. Only a few airborne measurements of larger sized particles exist today for specific situations [63,81–83]. Recently, airborne measurements were conducted in CV during the ASKOS/JATAC campaigns (2021/2022), in view of the validation of AEOLUS satellite data. Particles in the PAD range of 0.5–30 µm were collected using newly constructed giant particle collectors onboard a light aircraft and UAVs [84]. The results from this campaign are in progress and subject to future publications. It can be expected that the use of giant particle collectors will be useful in the near future for the detailed study of dust aerosol particles in a wider size spectrum.

## Data Availability

The datasets supporting this article have been uploaded as part of the electronic supplementary material [85]. Data for chemical composition and optical properties for all samples are uploaded as Excel files. The particle classification schemes used in the program are uploaded as electronic supplementary material2.

## References

[1] Prospero JM, Mayol-Bracero OL. 2013 Understanding the transport and impact of African dust on the Caribbean Basin. Bull. Am. Meteorol. Soc. **94**, 1329–1337. (10.1175/BAMS-D-12-00142.1)

[2] Carslaw KS, Boucher O, Spracklen DV, Mann GW, Rae JGL, Woodward S, Kulmala M. 2010 A review of natural aerosol interactions and feedbacks within the Earth system. Atmos. Chem. Phys. **10**, 1701–1737. (10.5194/acp-10-1701-2010)

[3] Prospero JM, Delany AC, Delany AC, Carlson TN. 2021 The discovery of African dust transport to the Western hemisphere and the saharan air layer: a history. Bull. Am. Meteorol. Soc. **102**, 1239–1260. (10.1175/BAMS-D-19-0309.1)

[4] Cuesta J, Marsham JH, Parker DJ, Flamant C. 2009 Dynamical mechanisms controlling the vertical redistribution of dust and the thermodynamic structure of the West saharan atmospheric boundary layer during summer. Atmos. Sci. Lett. **10**, 34–42. (10.1002/asl.207)

[5] Schepanski K, Tegen I, Macke A. 2009 Saharan dust transport and deposition towards the tropical northern Atlantic. Atmos. Chem. Phys. **9**, 1173–1189. (10.5194/acp-9-1173-2009)

[6] Schepanski K, Heinold B, Tegen I. 2017 Harmattan, Saharan heat low, and West African monsoon circulation: modulations on the Saharan dust outflow towards the North Atlantic. Atmos. Chem. Phys. **17**, 10223–10243. (10.5194/acp-17-10223-2017)

[7] Carlson TN, Prospero JM. 1972 The large-scale movement of Saharan air outbreaks over the Northern equatorial Atlantic. J. Appl. Meteor. **11**, 283–297. (10.1175/1520-0450(1972)011<0283:TLSMOS>2.0.CO;2)

[8] Tsamalis C, Chédin A, Pelon J, Capelle V. 2013 The seasonal vertical distribution of the saharan air layer and its modulation by the wind. Atmos. Chem. Phys. **13**, 11235–11257. (10.5194/acp-13-11235-2013)

[9] Kandler K *et al*. 2009 Size distribution, mass concentration, chemical and mineralogical composition and derived optical parameters of the boundary layer aerosol at Tinfou, Morocco, during SAMUM 2006. Tellus B. **61**, 32. (10.1111/j.1600-0889.2008.00385.x)

[10] Kandler K, Schneiders K, Heuser J, Waza A, Aryasree S, Althausen D, Hofer J, Abdullaev SF, Makhmudov AN. 2020 Differences and similarities of Central Asian, African, and Arctic dust composition from a single particle perspective. Atmosphere **11**, 269. (10.3390/atmos11030269)

[11] Klaver A *et al*. 2011 Physico‐chemical and optical properties of Sahelian and Saharan mineral dust: in situ measurements during the GERBILS campaign . Quart. J. Royal Meteoro. Soc. **137**, 1193–1210. (10.1002/qj.889)

[12] Formenti P, Caquineau S, Desboeufs K, Klaver A, Chevaillier S, Journet E, Rajot JL. 2014 Mapping the physico-chemical properties of mineral dust in western Africa: mineralogical composition. Atmos. Chem. Phys. **14**, 10663–10686. (10.5194/acp-14-10663-2014)

[13] Marticorena B, Haywood J, Coe H, Formenti P, Liousse C, Mallet M, Pelon J. 2011 Tropospheric aerosols over West Africa: highlights from the AMMA international program. Atmos. Sci. Lett. **12**, 19–23. (10.1002/asl.322)

[14] Heintzenberg J. 2009 The SAMUM-1 experiment over Southern Morocco: overview and introduction. Tellus B. **61**, 2. (10.1111/j.1600-0889.2008.00403.x)

[15] Ansmann A, Petzold A, Kandler K, Tegen I, Wendisch M, Müller D, Weinzierl B, Müller T, Heintzenberg J. 2011 Saharan Mineral Dust Experiments SAMUM–1 and SAMUM–2: what have we learned? Tellus B. **63**, 403. (10.1111/j.1600-0889.2011.00555.x)

[16] Reid EA, Reid JS, Meier MM, Dunlap MR, Cliff SS, Broumas A, Perry K, Maring H. 2003 Characterization of African dust transported to Puerto Rico by individual particle and size segregated bulk analysis. J. Geophys. Res. **108**. (10.1029/2002JD002935)

[17] Ryder CL *et al*. 2018 Coarse-mode mineral dust size distributions, composition and optical properties from AER-D aircraft measurements over the tropical eastern Atlantic. Atmos. Chem. Phys. **18**, 17225–17257. (10.5194/acp-18-17225-2018)

[18] van der Does M, Knippertz P, Zschenderlein P, Giles Harrison R, Stuut JBW. 2018 The mysterious long-range transport of giant mineral dust particles. Sci. Adv. **4**, eaau2768. (10.1126/sciadv.aau2768) 30547085 PMC6291315

[19] Adebiyi AA, Huang Y, Samset BH, Kok JF. 2023 Observations suggest that North African dust absorbs less solar radiation than models estimate. Commun. Earth Environ. **4**. (10.1038/s43247-023-00825-2)

[20] Di Biagio C *et al*. 2019 Complex refractive indices and single-scattering albedo of global dust aerosols in the shortwave spectrum and relationship to size and iron content. Atmos. Chem. Phys. **19**, 15503–15531. (10.5194/acp-19-15503-2019)

[21] Kok JF, Storelvmo T, Karydis VA, Adebiyi AA, Mahowald NM, Evan AT, He C, Leung DM. 2023 Mineral dust aerosol impacts on global climate and climate change. Nat. Rev. Earth Environ. **4**, 71–86. (10.1038/s43017-022-00379-5)

[22] Weinzierl B *et al*. 2017 The Saharan aerosol long-range transport and aerosol–cloud-interaction experiment: overview and selected highlights. Bull. Am. Meteorol. Soc. **98**, 1427–1451. (10.1175/BAMS-D-15-00142.1)

[23] Ginoux P. 2003 Effects of nonsphericity on mineral dust modeling. J. Geophys. Res. **108**. (10.1029/2002JD002516)

[24] Matsuki A, Schwarzenboeck A, Venzac H, Laj P, Crumeyrolle S, Gomes L. 2010 Cloud processing of mineral dust: direct comparison of cloud residual and clear sky particles during AMMA aircraft campaign in summer 2006. Atmos. Chem. Phys. **10**, 1057–1069. (10.5194/acp-10-1057-2010)

[25] Huang Y, Kok JF, Kandler K, Lindqvist H, Nousiainen T, Sakai T, Adebiyi A, Jokinen O. 2020 Climate Models and Remote Sensing Retrievals Neglect Substantial Desert Dust Asphericity. Geophys. Res. Lett. **47**. (10.1029/2019GL086592)

[26] Hinds WC, Zhu Y. 2022 Aerosol technology. properties, behavior, and measurement of airborne particles, Third edition. Hoboken NJ: Wiley.

[27] Kandler K et al. 2011 Electron microscopy of particles collected at Praia, Cape Verde, during the Saharan mineral dust experiment: particle chemistry, shape, mixing state and complex refractive index. Tellus B 63, 475. (10.1111/j.1600-0889.2011.00550.x).

[28] Kandler K, Benker N, Bundke U, Cuevas E, Ebert M, Knippertz P, Rodríguez S, Schütz L, Weinbruch S. 2007 Chemical composition and complex refractive index of Saharan Mineral dust at Izaña, Tenerife (Spain) derived by electron microscopy. Atmos. Environ. **41**, 8058–8074. (10.1016/j.atmosenv.2007.06.047)

[29] Fiebig M, Petzold A, Schröder S. 2002 The DLR falcon aerosol inlet - design and characteristics. Leipzig: EUFAR (European Fleet for Airborne Research).

[30] Kandler K, Schneiders K, Ebert M, Hartmann M, Weinbruch S, Prass M, Pöhlker C. 2018 Composition and mixing state of atmospheric aerosols determined by electron microscopy: method development and application to aged Saharan dust deposition in the Caribbean boundary layer. Atmos. Chem. Phys. 18, 13429–13455. (10.5194/acp-18-13429-2018).

[31] Panta A *et al*. 2023 Insights into the single-particle composition, size, mixing state, and aspect ratio of freshly emitted mineral dust from field measurements in the Moroccan Sahara using electron microscopy. Atmos. Chem. Phys. **23**, 3861–3885. (10.5194/acp-23-3861-2023)

[32] Walser A, Sauer D, Spanu A, Gasteiger J, Weinzierl B. 2017 On the parametrization of optical particle counter response including instrument-induced broadening of size spectra and a self-consistent evaluation of calibration measurements. Atmos. Meas. Tech. **10**, 4341–4361. (10.5194/amt-10-4341-2017)

[33] Stohl A, Wotawa G, Seibert P, Kromp-Kolb H. 1995 Interpolation errors in wind fields as a function of spatial and temporal resolution and their impact on different types of kinematic trajectories. J. Appl. Meteor. **34**, 2149–2165. (10.1175/1520-0450(1995)034<2149:IEIWFA>2.0.CO;2)

[34] Hinds WC. 1999 Aerosol technology. properties, behavior, and measurement of airborne particles, 2nd ed. Hoboken NJ: Wiley.

[35] Ott DK, Kumar N, Peters TM. 2008 Passive sampling to capture spatial variability in PM10-2.5. Atmos. Environ. **42**, 746–756. (10.1016/j.atmosenv.2007.09.058)

[36] Ott DK, Peters TM. 2008 A Shelter to Protect A Passive Sampler for Coarse Particulate Matter, PM 10-2.5 . Aerosol Sci. Technol. **42**, 299–309. (10.1080/02786820802054236)

[37] Bagheri G, Bonadonna C. 2016 On the drag of freely falling non-spherical particles. Powder Technol. **301**, 526–544. (10.1016/j.powtec.2016.06.015)

[38] Zhang XL, Wu GJ, Zhang CL, Xu TL, Zhou QQ. 2015 What is the real role of iron oxides in the optical properties of dust aerosols? Atmos. Chem. Phys. **15**, 12159–12177. (10.5194/acp-15-12159-2015)

[39] Caponi L *et al*. 2017 Spectral- and size-resolved mass absorption efficiency of mineral dust aerosols in the shortwave spectrum: a simulation chamber study. Atmos. Chem. Phys. **17**, 7175–7191. (10.5194/acp-17-7175-2017)

[40] Atkinson JD, Murray BJ, Woodhouse MT, Whale TF, Baustian KJ, Carslaw KS, Dobbie S, O’Sullivan D, Malkin TL. 2013 The importance of feldspar for ice nucleation by mineral dust in mixed-phase clouds. Nature **498**, 355–358. (10.1038/nature12278) 23760484

[41] Weinzierl B, Walser A, Dollner M. 2013 Daniel Sauer, and josef gasteiger size distribution data from the SALTRACE campaign. Dataset (10.25365/phaidra.308)

[42] Kandler K *et al*. 2011 Ground-based off-line aerosol measurements at Praia, Cape Verde, during the saharan mineral dust experiment: microphysical properties and mineralogy. Tellus B. **63**. (10.3402/tellusb.v63i4.16240)

[43] Formenti P *et al*. 2011 Recent progress in understanding physical and chemical properties of African and Asian mineral dust. Atmos. Chem. Phys. **11**, 8231–8256. (10.5194/acp-11-8231-2011)

[44] Barthelmy D. Mineralogy database. See http://www.webmineral.com.

[45] Caquineau S, Gaudichet A, Gomes L, Legrand M. 2002 Mineralogy of Saharan dust transported over northwestern tropical Atlantic Ocean in relation to source regions. J. Geophys. Res. **107**. (10.1029/2000JD000247)

[46] Groß S, Freudenthaler V, Schepanski K, Toledano C, Schäfler A, Ansmann A, Weinzierl B. 2015 Optical properties of long-range transported Saharan dust over barbados as measured by dual-wavelength depolarization raman lidar measurements. Atmos. Chem. Phys. **15**, 11067–11080. (10.5194/acp-15-11067-2015)

[47] Di Biagio C *et al*. 2017 Global scale variability of the mineral dust long-wave refractive index: a new dataset of in situ measurements for climate modeling and remote sensing. Atmos. Chem. Phys. **17**, 1901–1929. (10.5194/acp-17-1901-2017)

[48] Groß S, Tesche M, Freudenthaler V, Toledano C, Wiegner M, Ansmann A, Althausen D, Seefeldner M. Characterization of Saharan dust, marine aerosols and mixtures of biomass-burning aerosols and dust by means of multi-wavelength depolarization and Raman lidar measurements during SAMUM 2. Tellus B. **63**, 706. (10.1111/j.1600-0889.2011.00556.x)

[49] Glaccum RA, Prospero JM. 1980 Saharan aerosols over the tropical North Atlantic- mineralogy. Mar. Geol. **37**, 295–321. (10.1016/0025-3227(80)90107-3)

[50] Ma Q, He H, Liu Y, Liu C, Grassian VH. 2013 Heterogeneous and multiphase formation pathways of gypsum in the atmosphere. Phys. Chem. Chem. Phys. **15**, 19196–19204. (10.1039/c3cp53424c) 24107920

[51] Andreae MO, Charlson RJ, Bruynseels F, Storms H, VAN Grieken R, Maenhaut W. 1986 Internal mixture of sea salt, silicates, and excess sulfate in marine aerosols. Science **232**, 1620–1623. (10.1126/science.232.4758.1620) 17812139

[52] Sinha BW, Hoppe P, Huth J, Foley S, Andreae MO. 2008 Sulfur isotope analyses of individual aerosol particles in the urban aerosol at a central European site (Mainz, Germany). Atmos. Chem. Phys. **8**, 7217–7238. (10.5194/acp-8-7217-2008)

[53] Barkley AE *et al*. 2021 Atmospheric transport of north African dust‐bearing supermicron freshwater diatoms to South America: implications for iron transport to the equatorial North Atlantic Ocean. Geophys. Res. Lett. **48**. (10.1029/2020GL090476)

[54] Barkley AE *et al*. 2022 Interannual variability in the source location of North African dust transported to the Amazon. Geophys. Res. Lett. **49**. (10.1029/2021GL097344)

[55] Groß S, Gasteiger J, Freudenthaler V, Müller T, Sauer D, Toledano C, Ansmann A. 2016 Saharan dust contribution to the Caribbean summertime boundary layer – a lidar study during SALTRACE. Atmos. Chem. Phys. **16**, 11535–11546. (10.5194/acp-16-11535-2016)

[56] Huang Y, Adebiyi AA, Formenti P, Kok JF. 2021 Linking the different diameter types of aspherical desert dust indicates that models underestimate coarse dust emission. Geophys. Res. Lett. **48**. (10.1029/2020GL092054)

[57] Mayer B, Kylling A. 2005 Technical note: The libRadtran software package for radiative transfer calculations - description and examples of use. Atmos. Chem. Phys. **5**, 1855–1877. (10.5194/acp-5-1855-2005)

[58] Drakaki E et al. 2022 Modeling coarse and giant desert dust particles. Atmos. Chem. Phys. 22, 12727–12748. (10.5194/acp-22-12727-2022).

[59] Haynes WM. 2014 CRC handbook of chemistry and physics. Boca Raton, FL: CRC Press.

[60] Schladitz A, Müller T, Nordmann S, Tesche M, Groß S, Freudenthaler V, Gasteiger J, Wiedensohler A. 2011 In situ aerosol characterization at Cape Verde. Tellus B. Chem. Phys. Meteorol. **63**, 549–572. (10.1111/j.1600-0889.2011.00568.x)

[61] Solmon F, Mallet M, Elguindi N, Giorgi F, Zakey A, Konaré A. 2008 Dust aerosol impact on regional precipitation over western Africa, mechanisms and sensitivity to absorption properties. Geophys. Res. Lett. **35**. (10.1029/2008GL035900)

[62] Denjean C *et al*. 2016 Size distribution and optical properties of African mineral dust after intercontinental transport. J. Geophys. Res. **121**, 7117–7138. (10.1002/2016JD024783)

[63] McConnell CL, Formenti P, Highwood EJ, Harrison MAJ. 2010 Using aircraft measurements to determine the refractive index of Saharan dust during the DODO Experiments. Atmos. Chem. Phys. **10**, 3081–3098. (10.5194/acp-10-3081-2010)

[64] Osborne SR, Baran AJ, Johnson BT, Haywood JM, Hesse E, Newman S. 2011 Short‐wave and long‐wave radiative properties of Saharan dust aerosol. Quart. J. Royal Meteoro. Soc. **137**, 1149–1167. (10.1002/qj.771)

[65] Haywood JM *et al*. 2008 Overview of the dust and biomass‐burning experiment and African monsoon multidisciplinary analysis special observing period‐0. J. Geophys. Res. **113**. (10.1029/2008JD010077)

[66] Lieke K *et al*. 2011 Particle chemical properties in the vertical column based on aircraft observations in the vicinity of Cape Verde Islands. Tellus B. **63**, 497. (10.1111/j.1600-0889.2011.00553.x)

[67] Müller T, Schladitz A, Kandler K, Wiedensohler A. Spectral particle absorption coefficients, single scattering albedos and imaginary parts of refractive indices from ground based in situ measurements at Cape Verde Island during SAMUM-2. Tellus B. **63**, 573. (10.1111/j.1600-0889.2011.00572.x)

[68] Scheuvens D, Schütz L, Kandler K, Ebert M, Weinbruch S. 2013 Bulk composition of northern African dust and its source sediments -a compilation. Earth Sci. Rev. **116**, 170–194. (10.1016/j.earscirev.2012.08.005)

[69] Bozlaker A, Prospero JM, Price J, Chellam S. 2018 Linking barbados mineral dust aerosols to North African sources using elemental composition and radiogenic Sr, Nd, and Pb Isotope signatures. J. Geophys. Res. **123**, 1384–1400. (10.1002/2017JD027505)

[70] Aryasree S, Nair PR, Girach IA, Jacob S. 2015 In situ measured seasonal characteristics of near‐surface aerosols over Bay of Bengal and MODIS‐retrieved columnar properties: a multicampaign analysis. J. Geophys. Res. **120**. (10.1002/2015JD023418)

[71] Arnold E, Merrill J, Leinen M, King J. 1998 The effect of source area and atmospheric transport on mineral aerosol collected over the North Pacific Ocean. Glob. Planet. Change **18**, 137–159. (10.1016/S0921-8181(98)00013-7)

[72] Gasteiger J, Groß S, Sauer D, Haarig M, Ansmann A, Weinzierl B. Particle settling and vertical mixing in the Saharan air layer as seen from an integrated model, lidar, and in situ perspective. Atmos. Chem. Phys. **17**, 297–311. (10.5194/acp-17-297-2017)

[73] Reid JS *et al*. 2003 Analysis of measurements of Saharan dust by airborne and ground‐based remote sensing methods during the puerto rico dust Experiment (PRIDE). J. Geophys. Res. **108**. (10.1029/2002JD002493)

[74] Reid JS *et al*. 2003 Comparison of size and morphological measurements of coarse mode dust particles from Africa. J. Geophys. Res. **108**. (10.1029/2002JD002485)

[75] Shi Z, Krom MD, Jickells TD, Bonneville S, Carslaw KS, Mihalopoulos N, Baker AR, Benning LG. 2012 Impacts on iron solubility in the mineral dust by processes in the source region and the atmosphere: a review. Aeolian Res. **5**, 21–42. (10.1016/j.aeolia.2012.03.001)

[76] Groß S *et al*. 2011 Characterization of the planetary boundary layer during SAMUM-2 by means of lidar measurements. Tellus B. **63**, 695. (10.1111/j.1600-0889.2011.00557.x)

[77] Menut L, Siour G, Bessagnet B, Couvidat F, Journet E, Balkanski Y, Desboeufs K. 2020 Modelling the mineralogical composition and solubility of mineral dust in the mediterranean area with CHIMERE 2017r4. Geosci. Model Dev. **13**, 2051–2071. (10.5194/gmd-13-2051-2020)

[78] Gonçalves Ageitos M *et al*. 2023 Modeling dust mineralogical composition: sensitivity to soil mineralogy atlases and their expected climate impacts. Atmos. Chem. Phys. **23**, 8623–8657. (10.5194/acp-23-8623-2023)

[79] Nousiainen T, Kandler K. 2015 Light scattering by atmospheric mineral dust particles. In Light scattering (ed. AA Kokhanovsky), pp. 3–52. Berlin: Heidelberg: springer Berlin Heidelberg. (10.1007/978-3-642-37985-7_1)

[80] Arreyndip NA, Kandler K, Sudharaj A. 2023 Near-field single-scattering calculations of aerosols: sensitivity studies. Optics **4**, 375–395. (10.3390/opt4020028)

[81] Levin Z, Teller A, Ganor E, Yin Y. 2005 On the interactions of mineral dust, sea‐salt particles, and clouds: a measurement and modeling study from the mediterranean Israeli dust experiment campaign. J. Geophys. Res. **110**. (10.1029/2005JD005810)

[82] Price HC *et al*. 2018 Atmospheric ice‐nucleating particles in the dusty tropical atlantic. J. Geophys. Res. **123**, 2175–2193. (10.1002/2017JD027560)

[83] Schumann U *et al*. 2011 Airborne observations of the eyjafjalla volcano ash cloud over Europe during air space closure in april and may 2010. Atmos. Chem. Phys. **11**, 2245–2279. (10.5194/acp-11-2245-2011)

[84] Kezoudi M *et al*. 2021 The unmanned systems research laboratory (USRL): a new facility for UAV-based atmospheric observations. Atmos. Basel. **12**, 1042. (10.3390/atmos12081042)

[85] Sudharaj A *et al*. 2024 Supplementary material from: vertical variability in morphology, chemistry, and optical properties of the transported saharan air layer measured from cape verde and the caribbean. Figshare (10.6084/m9.figshare.c.7468078) PMC11539264 39507994

